# Allergenicity and structural properties of new Cor a 1 isoallergens from hazel identified in different plant tissues

**DOI:** 10.1038/s41598-024-55856-2

**Published:** 2024-03-07

**Authors:** Julian M. Hendrich, Andreas Reuter, Thessa P. Jacob, Hatice Kara, Sherine Amer, Katharina Rödel, Birgitta M. Wöhrl

**Affiliations:** 1 Lehrstuhl Biochemie IV - Biophysikalische Chemie, Universität Bayreuth, Universitätsstrasse 30, 95447 Bayreuth, Germany; 2 Division of Allergology, Paul-Ehrlich-Institut, 63225 Langen, Germany

**Keywords:** PR-10 protein, Bet v 1, Cor a 1 isoallergens, Gene expression, LC-MS^E^, Hazel genome, Corylus avellana, Immunoblot, ELISA, RT-PCR, T_m_-values, Biochemistry, Biophysics, Immunology, Molecular biology, Plant sciences, Structural biology

## Abstract

The hazel allergen Cor a 1 is a PR-10 protein, closely related to the major birch pollen allergen Bet v 1. Hazel allergies are caused by cross-reactive IgE antibodies originally directed against Bet v 1. Despite the importance of PR-10 proteins in allergy development, their function and localization in the plant remain largely elusive. Therefore, the presence of Cor a 1 mRNA and proteins was investigated in different tissues, i.e., the female flower, immature and mature nuts, catkins, and pollen. Four yet unknown Cor a 1 isoallergens, i.e., Cor a 1.0501–1.0801, and one new Cor a 1.03 variant were discovered and characterized. Depending on the isoallergen, the occurrence and level of mRNA expression varied in different tissues, suggesting different functions. Interestingly, Cor a 1.04 previously thought to be only present in nuts, was also detected in catkins and pollen. The corresponding Cor a 1 genes were expressed in *Escherichia coli*. The purified proteins were analysed by CD and NMR spectroscopy. Immunoblots and ELISAs to determine their allergenic potential showed that the new proteins reacted positively with sera from patients allergic to birch, hazel and elder pollen and were recognized as novel isoallergens/variants by the WHO/IUIS Allergen Nomenclature Sub-Committee.

## Introduction

Anemophilous or wind-driven pollen from certain trees can trigger allergic diseases and therefore impose serious health problems. An estimated 10–30% of the global population are afflicted by seasonal allergies and asthma caused by pollen, and the number continues to rise^1^. Changes in life style, environmental pollution or dietary habits as well as climate change appear to play a role^2^. One of the best studied allergens is the major birch pollen allergen Bet v 1, which is the founding member of the Bet v 1-like superfamily of proteins. This superfamily has representatives in all three domains of life, archeae, bacteria and eukaryotes^3^, and is comprised of 11 subfamilies. Bet v 1 itself belongs to the subfamily of the pathogenesis related proteins 10 (PR-10)-like family of proteins^3–5^. Most of the Bet v 1 homologous allergens from pollen and plants, including the Cor a 1 allergen from hazel, belong to this subfamily. Whereas “real” PR-10 proteins, which have been detected in various plants are only induced in pathological stress situations, suggesting a role in plant defence responses, PR-10-like proteins are constitutively expressed, indicating a more general role in plant development^3,6,7^.

In most cases allergic reactions to Bet v 1 and homologous proteins are not life threatening and usually result in mild symptoms like rhinitis, burning and itching of the tongue and oral mucosa, which are described as the so-called oral allergy syndrome (OAS) or pollen food syndrome (PFS). However, allergy induced asthma can also occur^4,8–10^. Most pollen allergy sufferers are primarily sensitized to Bet v 1^11^. Over time, many patients develop cross-reactivity of IgE antibodies originally directed against Bet v 1 proteins to homologous PR-10 allergens, which are found in almost all angiosperms and have at least 50% sequence identity amongst them^6^. Thus, birch pollen allergic individuals frequently experience IgE mediated allergic reactions upon contact with various plant food products harbouring homologous PR-10 proteins, i.e. fruits like apple, cherry or peach, vegetables and roots such as celeriac or carrots, and nuts, e.g. almonds and hazelnuts^4,12^.

The structural similarity of PR-10 allergens is the basis of the development of cross-reactive IgE antibodies^13^. The three-dimensional structure of many PR-10 and PR-10-like proteins is known and is almost identical^6^. They exhibit conserved, common structural elements, suggesting a general and maybe indispensable function in plants. They consist of a seven-stranded, antiparallel β-sheet, a long C-terminal α-helix and two short helices arranged in V-shape, which together form a predominantly hydrophobic or amphiphilic pocket, being composed of a combination of hydrophilic and hydrophobic residues, that can bind small ligands^14–18^. A variety of small molecules were shown to bind to different PR-10 allergens, e.g. flavonoids, steroids or cytokinins, suggesting that they are involved in transport and/or storage of secondary plant compounds, UV protection and germination^15,17,19–23^. However, for most PR-10 allergens the precise functions in the plant remain elusive.

IgE antibodies of Bet v 1 allergic individuals can also cross-react with the corresponding Cor a 1 allergen from hazel (*Corylus avellana*)^24,25^. Consequently, contact with hazel pollen can trigger an allergic reaction similar to that described above, and in addition, hazelnut is a common cause of food allergy.^10,25–28^.

Due to the high relevance for the development of cross-allergies, it is imperative to obtain a more complete picture of the occurrence of known and novel Cor a 1 isoallergens in different hazel tissues. So far, four Cor a 1 isoallergens (> 67% amino acid sequence identity), termed Cor a 1.01 to Cor a 1.04, have been identified^29,30^. Four different Cor a 1.01 variants (> 90% identity), named Cor a 1.0101 to Cor a 1.0104, have been found in hazel pollen^29^. Furthermore, four variants of the isoallergen Cor a 1.04 have been detected in nuts^30^. In contrast, Cor a 1.02 and Cor a 1.03 have been suggested to occur in leave tissue, however in that work the corresponding genes were only detected in the genome, but no expression analyses, neither on the mRNA nor on the protein level, were performed^31^. Thus, the presence of those isoallergens in different hazel tissues and in pollen has not yet been confirmed.

A recent study produced a fully assembled genome sequence and annotation for the hazelnut species *C. avellana* cv ‘Tombul’, an important Turkish variety. In addition to the known Cor a 1 genes, several new Cor a 1 related sequences were detected, some of which with only 40–50% identity to Cor a 1^32^. The study implies that there are many more than the already known Cor a 1 isoallergens and variants. However, similar to most other PR-10 allergens, the exact functions and localization of Cor a 1 proteins in the plant are not known. Some of the Cor a 1 proteins appear to be involved in transport and/or storage of flavonoids. We have previously identified a natural ligand of the Cor a 1.04 isoallergen, quercetin-3-O-(2“-O-β-d-glucopyranosyl)-β-Dgalactopyranoside (Q3O-(Glc)-Gal), and suggested a function of the protein/ligand complex in fertilization^17^.

As mentioned above, some Cor a 1 proteins have been shown to be localized in pollen or nuts^29^. However, no comprehensive study has yet been conducted to demonstrate which Cor a 1 proteins are present in different plant tissues. Therefore, we wanted to identify new Cor a 1 isoallergens and analyse their biochemical and biophysical properties as well as the allergenic potential to contribute to a better understanding of hazel allergy.

## Results and discussion

### Identification and mRNA expression profiles of Cor a 1 isoallergens

There appear to be more Cor a 1 isoallergens and variants present in hazel than have been described so far, as indicated by similarities of genes found in the hazel genome^32^. Although the presence of the isoallergens Cor a 1.01 and Cor a 1.04 in pollen and kernel, respectively, has been shown, the expression and localization of the other known isoallergens Cor a 1.02 and Cor a 1.03 in different tissues has not yet been clarified.

To shed more light on the occurrence and distribution of Cor a 1 isoallergens, we determined the presence of Cor a 1 mRNA in different hazel plant tissues and in pollen. Since the fully assembled and annotated genome sequence of a European hazel species, *C. avellana* cv ‘Tombul’ was recently published^32^ it was possible to search specifically for novel Cor a 1 genes and their expression in different hazel tissues.

**Figure 1:**
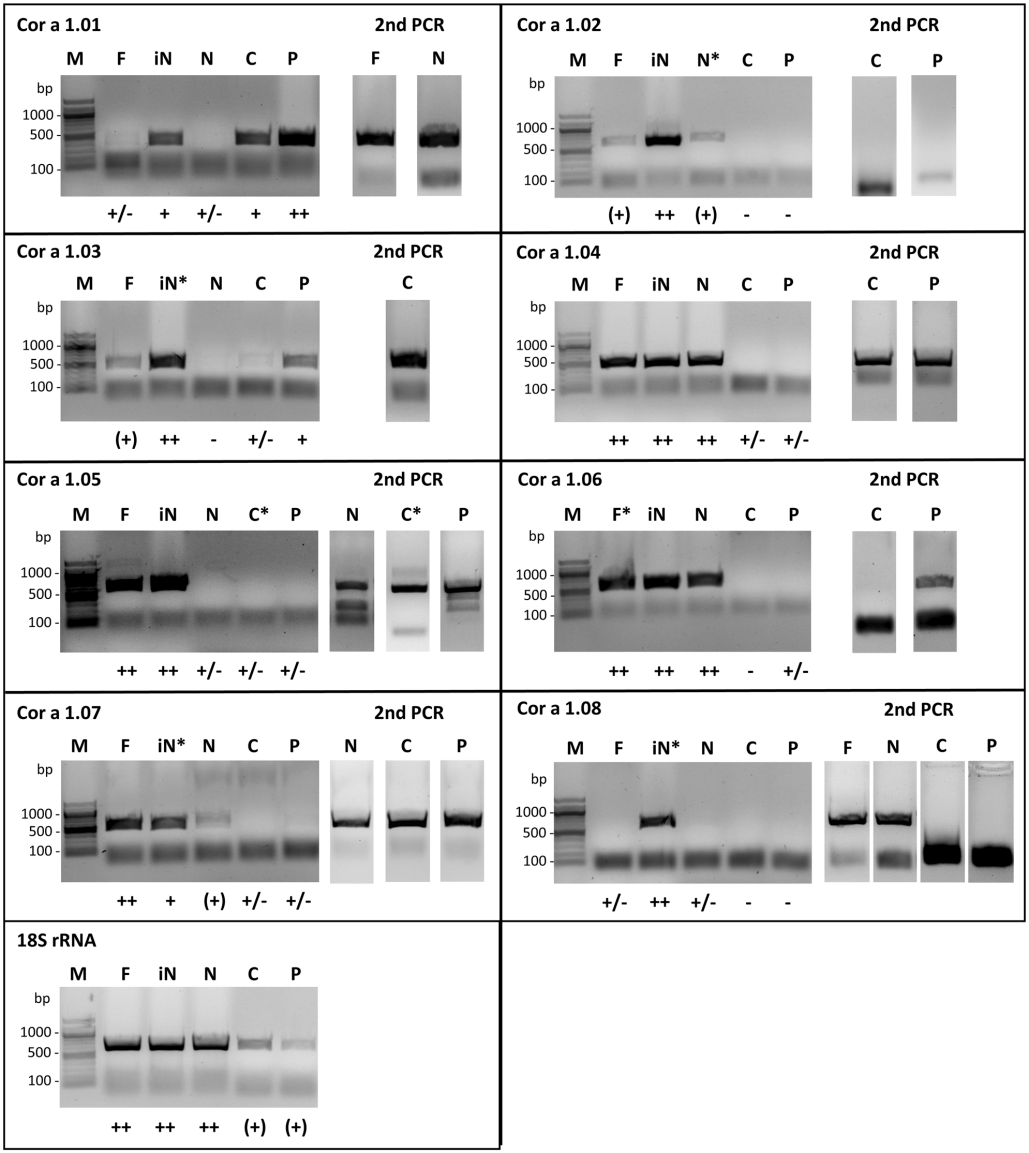
Expression of Cor a 1 genes in different hazel tissues. The RT-PCR products using gene specific primers to determine Cor a 1 mRNA expression were loaded on 1.5% agarose gels. In case no band was visible, a gel slice at the corresponding height was cut out. The purified mRNA was then used for the 2nd PCR. F: female flower; iN: immature nut; N: mature nut; C: catkin; P: pollen; ((+)) very low, (+): low, +: moderate, ++: strong expression levels. The RT-PCR samples used for cloning of the genes into expression vectors are marked with *M: 100 bp DNA standard (New England Biolabs; Frankfurt, Germany). 18S rRNA isolated in a similar fashion was used as a control.

Total RNA was isolated from the female flower, immature and mature nuts, male catkins, and pollen. After cDNA production with an mRNA-specific poly(T) primer, PCR was performed using specific primers binding either to the 5′ or 3′ end of the gene (only Cor a 1.0302) or to the 5′ and 3′ untranslated regions (UTRs). Since the UTR regions of variants of a certain isoallergen are rather similar or even identical^32^, it was not possible to distinguish different variants using this method. Thus, only the existence of a specific isoallergen, possibly consisting of several variants, could be determined in a specific plant material (Figs. 1, S6). However, this procedure allowed us to use the PCR sample for cloning and for sequencing the complete amplified gene for the identification of new isoallergens (Table S1). To verify that the amplificates contained Cor a 1 DNA, all PCR probes were subjected to DNA sequencing. To detect even extremely low specific mRNA levels, a 2nd PCR amplification step was carried out in such cases with the gel purified DNA of the first PCR.

So far, the Cor a 1.01 protein has been detected in hazel pollen^29^. However, the mRNA expression analyses presented here indicate that Cor a 1.01 is, in addition, expressed in minimal amounts in the female flower and in the mature nut as indicated by the 2nd PCR amplification step. Significantly stronger specific mRNA levels could be detected in the immature nut as well as in catkins and pollen.

Cor a 1.02 mRNA was present in the female flower and the mature nut, and high mRNA concentrations were visible in the immature nut, but no specific mRNA was discovered in the male tissues. Moreover, a new, putative Cor a 1.02 variant, designated Cor a 1.02- Cav01g11820 was detected via sequencing of the PCR product (Figure S1). However, since it was not possible to purify the protein from *E. coli* to determine its allergenic properties, this variant was not considered further in our studies.

Cor a 1.03 mRNA was present in all tissues with the exception of the mature nut even though high mRNA levels were detected in the immature nut. Although we could detect a very faint band after the first PCR round of the mature nut, sequencing of the amplificate of the 2^nd^ PCR revealed no Cor a 1.03 gene sequence (Fig. 1). In addition, using LC-MS^E^ (liquid chromatography–mass spectrometry^elevated energy^) variants of Cor a 1.01 and Cor a 1.03 could also be discovered on the protein level by LC-MS^E^ in pollen extract (Figure S2).

It has been shown previously that Cor a 1.04 is present in nuts^30^. Since Cor a 1.04 was found to bind to a ligand isolated from pollen extracts it was suggested that it might play a role in fertilization^17^. Thus, it was interesting to determine whether Cor a 1.04 is localized in other tissues as well. Surprisingly, the 2nd PCR amplification step followed by sequencing revealed the presence of Cor a 1.04 specific mRNA also in catkins and pollen, albeit at very low amounts (Fig. 1). Thus, we show for the first time that Cor a 1.04 expression also occurs in the male tissues. This result provides a reasonable explanation why a ligand isolated from pollen extracts bound specifically to Cor a 1.0401 which was thought to be only present in the nut.

In addition, Cor a 1.04 mRNA could also be detected in the female flower and the immature nut (Fig. 1). Furthermore, LC-MS^E^ data showed the presence of the Cor a 1.04 protein in extracts of the mature nut (Figure S2). However, only a few peptides could be confirmed, suggesting that protein isolation from nuts might be less efficient than mRNA extraction. In addition, no amplification step is performed before the MS analysis. This might also explain why other isoallergens (Fig. 1) could not be identified at the protein level by LC-MS^E^. Moreover, it is also possible that protein amounts in nuts are lower and do not necessarily correspond to mRNA levels.

Cor a 1.05 mRNA was found in the female flower and in the immature nut, however, in the mature nut, as well as in catkins and pollen specific mRNA was only visible after the 2nd PCR procedure (Fig. 1).

Cor a 1.06 mRNA was detected in the female flower, as well as in the immature and mature nut, and was also visible in pollen after the 2nd PCR. Cor a 1.07 mRNA levels were very low in all tissues. In contrast, Cor a 1.08 mRNA concentration was high only in the immature nut, whereas it was weak in the female flower and mature nut, and no expression could be verified in the male tissues (Fig. 1).

It is important to note, that our intention was to identify new expressed Cor a 1 genes by detecting the corresponding mRNAs in different tissues and to clone the genes. Our data represent only one specific time point, which means that Cor a 1 isoallergens that were not visible in our experiments might nevertheless be present at an earlier or later stage of development.

The observation that the mRNA expression of the individual isoallergens differs so strongly implies different properties and functions in the plant, e.g. during fertilization and kernel development, that still need to be explored. Different roles of Cor a 1 isoallergens have already been suggested as only the Cor a 1.04 isoallergen but not Cor a 1.01 binds the flavonoid derivative Q3O-(Glc)-Gal^17^. In summary, the data show that there are more Cor a 1 isoallergens than previously known and that their presence in mature nuts and pollen could play a role in hazelnut allergy (see below).

### Identification of additional variants

In order to identify new isoallergens and variants, the amplified DNA products from specific tissues were cloned into the pCR-Blunt vector (Thermo Fisher, Schwerte, Germany) and sequenced. It turned out that several highly similar variants of a certain isoallergen could be detected in the different tissues tested (Figures S1, and S3). For some of them, only gene fragments could be amplified (data not shown). Due to the high similarity of the variants only one variant per isoallergen was selected and included in our studies to verify the biochemical and biophysical properties as well as the allergenicity (Figure S1). Furthermore, we identified variants of several isoallergens that could not be detected in the genome of the Turkish cultivar “Tombul”^32^ by BLAST analyses, indicating genetic differences of *C. avellana* used in our studies. These genes are labelled with a GenBank accession number only, whereas a Cav01 number represents genes also found in the “Tombul cultivar”. Our data indicate that for each isoallergen additional potential variants are present (Figure S3). However, due to their high similarity we did not include different variants of a certain isoallergen in our study.

As described above, we were able to identify four new isoallergens, namely Cor a 1.0501, Cor a 1.0601, Cor a 1.0701 and Cor a 1.0801 on the mRNA level (Figure S1). In addition to those isoallergens a new Cor a 1.03 variant, designated Cor a 1.0302 was detected. These proteins were included in biophysical and immunological studies. Due to their allergenic potential (see below), the four new isoallergens were recognized by the WHO/IUIS Allergen Nomenclature Sub-Committee and named as described.

Amino acid sequence alignments and the identity matrix of Cor a 1 isoallergens and variants revealed that the new proteins possess sequence identities to the already known isoallergens of 46.88 – 84.38%. The new Cor a 1.0302 variant is 96.23% identical to Cor a 1.0301 (Table 1).

**Table 1:**
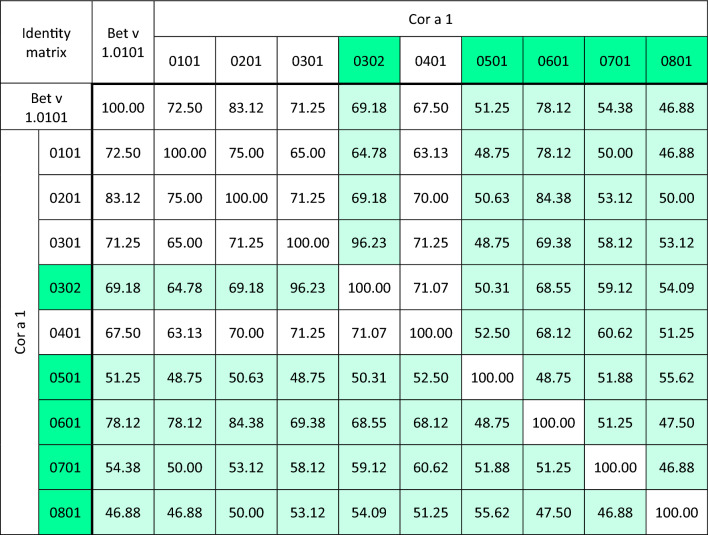
Identity matrix of Cor a 1 isoallergens and variants.

Numbers indicate the amino acid sequence identity in %

### Biochemical and biophysical analyses

The identified genes were cloned into the expression vectors pETGB1a or pET11a (only Cor a 1.0302) and expressed in *E. coli* (Table S2)*.* The SDS polyacrylamide gel of the purified proteins exhibits apparent molecular masses of ca. 16 kDa to 18 kDa (Figure S4).

**Figure 2:**
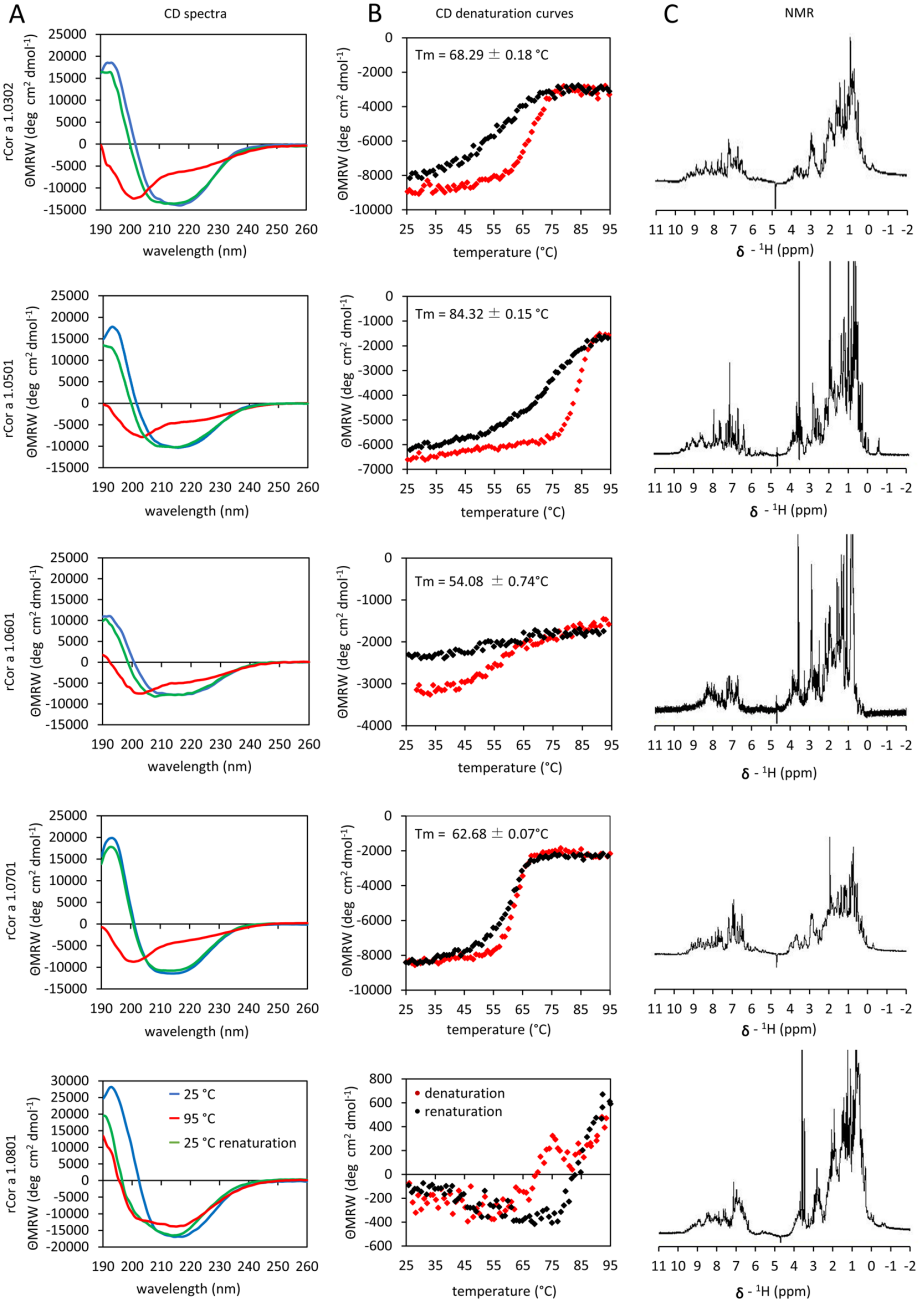
Physicochemical characterization of Cor a 1 isoallergens. (**A**) CD spectroscopy. CD spectra at 25 °C (blue curve), after heating to 95 °C (red curve), followed by stepwise cooling to 25 °C (green curve). (**B**) CD melting curves. CD denaturation (red) and renaturation (black) curves were recorded at 217 or 220 nm in 10 mM Na_2_HPO_4_/NaH_2_PO_4_ pH 7.0. The T_m_ value is displayed on top of each spectrum. No Tm could be calculated for Cor a 1.0801. (**C**) ^1^H-NMR-spectroscopy. Spectra of Cor a 1.0302 (160 µM), Cor a 1.0501 (200 µM), Cor a 1.0601 (60 µM), Cor a 1.0701 (190 µM) and Cor a 1.0801 (70 µM) were recorded in a Bruker Avance 600 MHz spectrometer at 295 K in 10% (v/v) D_2_O, 10 mM Na_2_HPO_4_/NaH_2_PO_4_, pH7.0.

To determine the refolding capacity and stability of the proteins, CD spectra were recorded (Fig. 2, panel A, B). At 25 °C the spectra of the recombinant (r)Cor a 1 isoallergens are characteristic for proteins harboring α- and β-like secondary structures (Fig. 2, panel A). After heating to 95 °C, rCor a 1.0302, 1.0501, 1.0601, and 1.0701 unfolded as indicated by the minimum at 200 nm. In contrast, rCor a 1.0801 appeared to be more stable. The curve after heating to 95 °C (Fig. 2, panel A, red curve) changed only slightly and there was no discrete minimum at 200 nm, signifying that only a few sections of the protein became unfolded. After cooling to 25 °C, rCor a 1.0302, 1.0501, 1.0601 and 1,0701 almost completely refolded whereas rCor a 1.0801 was still folded and the proportion of unfolded protein moieties decreased as compared to the spectrum at 95 °C (Fig. 2, panel A, green curves).

rCor a 1.0302, 1.0601 and 1.0701 revealed relatively similar T_m_-values of 68.29, 54.08 or 62.68 °C. Cor a 1.0501 exhibited the highest T_m_-value of 84 °C (Fig. 2, panel B). rCor a 1.0801 displayed a biphasic melting behaviour during heating to 95 °C. However, complete denaturation could not be achieved. Due to the small differences of the denaturation and renaturation curves, the noise in the measurement of the melting curve of Cor a 1.0801 is quite high (Fig. 2, panel B).

Cor a 1.0801 shows a lower sequence identity to Bet v 1.0101 and also to most of the other Cor a 1 isoallergens (Table 1). This difference could be the reason for its higher thermostability. Previously, the structure of the hypothetical protein TTHA0849 from the thermophilic bacterium *Thermus thermophilus* had been determined, which also shows the typical PR-10 fold of Bet v 1^33^. However, sequence comparisons revealed no evidence of conserved regions in Cor a 1.0801 and TTHA0849 that might explain its thermostability. Despite the similar three-dimensional structures, the sequence identity between Cor a 1.08 and TTHA0849 is only 20.98%, which is even lower than that of all the other Cor a 1 isoallergens to TTHA0849, except Cor a 1.05 (20.42%) (Figure S7).

While CD spectra can be used to determine secondary structures, 1D NMR spectroscopy can reveal three-dimensional folding of a protein. The 1D NMR spectra showed dispersion of the amide proton signals and high-field shifted signals of the methyl groups, which confirmed a three-dimensional folding for all isoallergens (Fig. 2, panel C).

These data suggest that the high stability and refolding capacity of the proteins could have an impact on their allergenicity. Heating might not completely abolish the allergenicity in hazel nut containing food stuff as Cor a 1 proteins could refold if the food is cooled down.

### IgE binding

**Figure 3:**
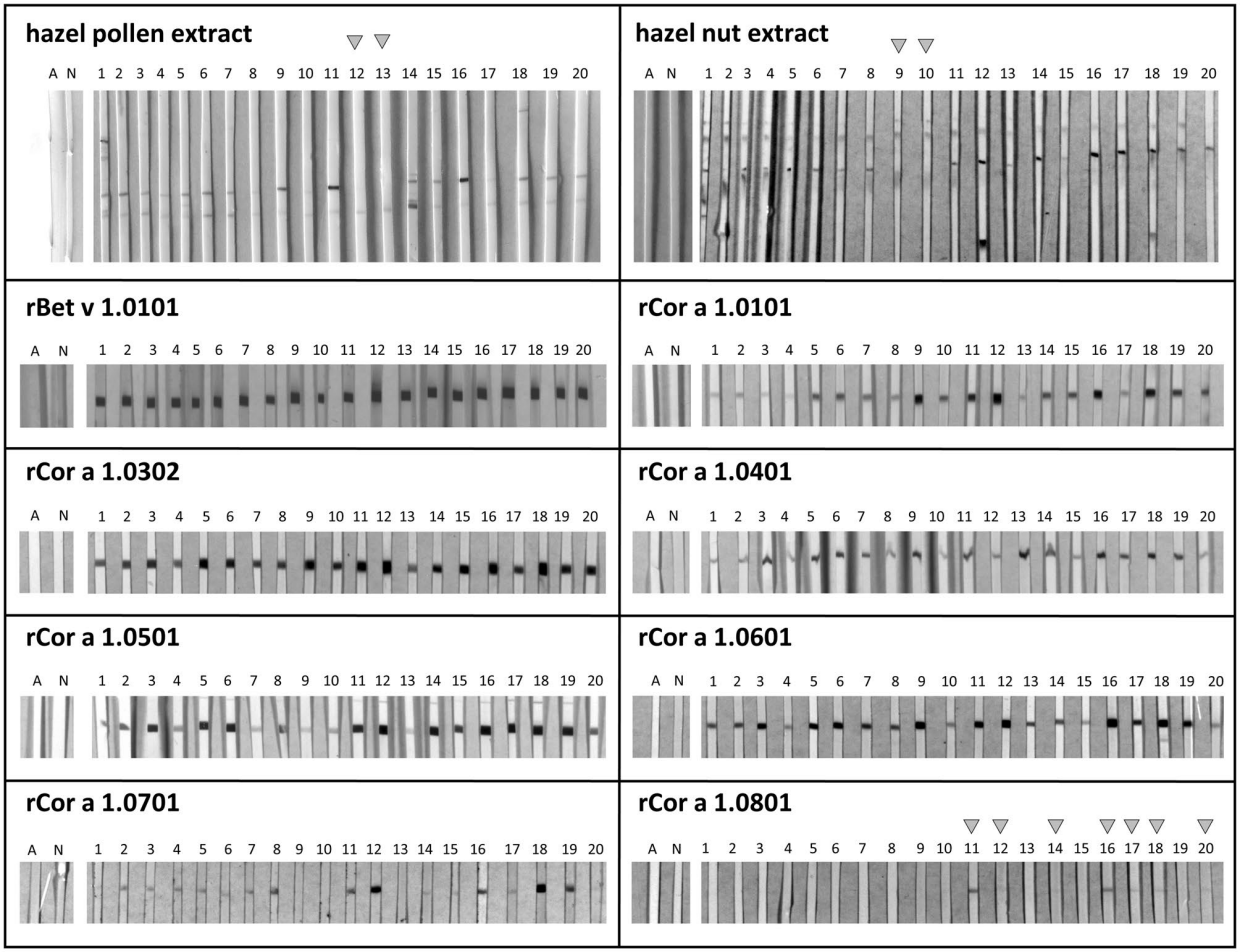
Immunoblots. SDS-PAGE (16% PA gels) was performed with the Cor a 1 proteins, followed by blotting onto a 0.2 µm nitrocellulose membrane as indicated on top of the immunoblots. 1.5 µg/cm protein was blotted except for Cor a 1.0801 with 0.8 µg/cm. The membranes were cut into stripes and incubated with patients’ sera. Bound specific IgE antibodies were detected using a mouse-anti-human IgE antibody coupled to alkaline phosphatase followed by nitroblue tetrazoliumchloride (NBT)/5-Bromo-4-chloro-3-indolylphosphate (BCIP) staining. The grey arrows indicate weak IgE binding for nCor a 1 from hazel pollen and hazel nut extract and for rCor a 1.0801. Labels on top of the immunoblots: A: secondary antibody control; N: horse serum; 1–20: sera of patients allergic against tree pollen.

The rCor a 1 proteins purified from *E. coli* were further used for immunological studies in order to determine their allergenicity. Sera from 20 tree-pollen allergic individuals that had been tested positive for specific IgE (Spez. IgE Kit from Gold Standard Diagnostics, Kassel, Germany) against a mixture of birch, hazel and alder pollen were used for immunoblot analyses with the recombinant isoallergens. Only sera reaching values ≥ 3.5 U/ml corresponding to the enzyme-allergo-sorbent-test (EAST) class 3 or higher were selected. As expected, all sera reacted strongly with the Bet v 1 control from birch since this is usually the sensitizing agent^4^ (Fig. 3, Figure S5). Furthermore, all sera reacted with rCor a 1.0101, albeit with varying intensities. Similarly, IgE binding could be detected with all sera employing rCor a 1.0302, 1.0401, 1.0501, and 1.0601, indicating that all of them are potent allergens. Using rCor a 1.0701, serum #1, #9, #10, and #13 displayed no IgE binding. rCor a 1.0801 proved to be the weakest allergen and showed IgE binding only with seven sera, namely #11, #12, #14, #16, #17, #18, and #20. Obviously, the sera responded better to those Cor a 1 isoallergens which possess a high identity with the sensitizing Bet v 1.0101 protein (Table 1). The weak allergens Cor a 1.0701 and Cor a 1.0801 have only 54.38 and 46.88% identity with Bet v 1.0101, respectively.

Since we have previously described that denaturation of an allergen during the blotting procedure can impair IgE binding^34^, ELISAs were chosen as an additional method to confirm the allergenicity of the isoallergens (Table 2). Here, all sera reacted with rCor a 1.0101 to rCor a 1.0601. With the exception of serum #12, all sera appeared to be less reactive using Cor a 1.0701. However, in contrast to the Western Blots, rCor a 1.0701 exhibited IgE binding with all sera, indicating that denaturation of the antigen in the Blots could play a role. Furthermore, the ELISA might be more sensitive than the Blot. Interestingly serum #12 was highly reactive with all isoallergens other than Cor a 1.0801. In addition to the sera reacting with Cor a 1.0801 in the above Western Blot, Cor a 1.0801 also showed weak IgE binding using serum #19. These results confirm that Cor a 1.0801 is a weaker allergen than the others but nevertheless several individuals appear to possess reactive IgE antibodies.

**Table 2:**
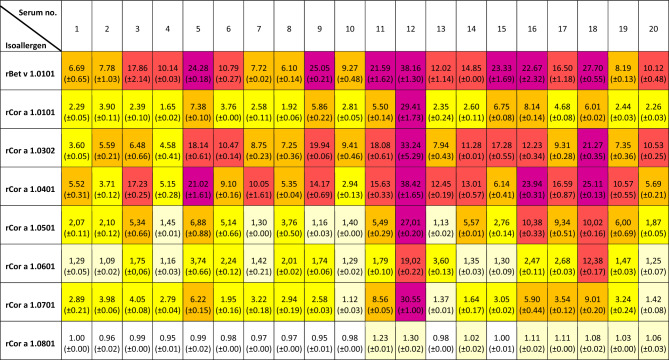
ELISA with purified rCor a 1 isoallergens and sera of tree pollen allergic patients.

The values represent the ratio of the measurements at 450/620 nm normalized to the 450/620 ratio of the negative control (horse serum). Serum dilution 1:10. Two technical replicates were performed.

Light yellow: 1–1.5, yellow: 1.5–5, orange: 5–10, red: 10–20, purple: > 20.

## Concluding remarks

**Figure 4:**
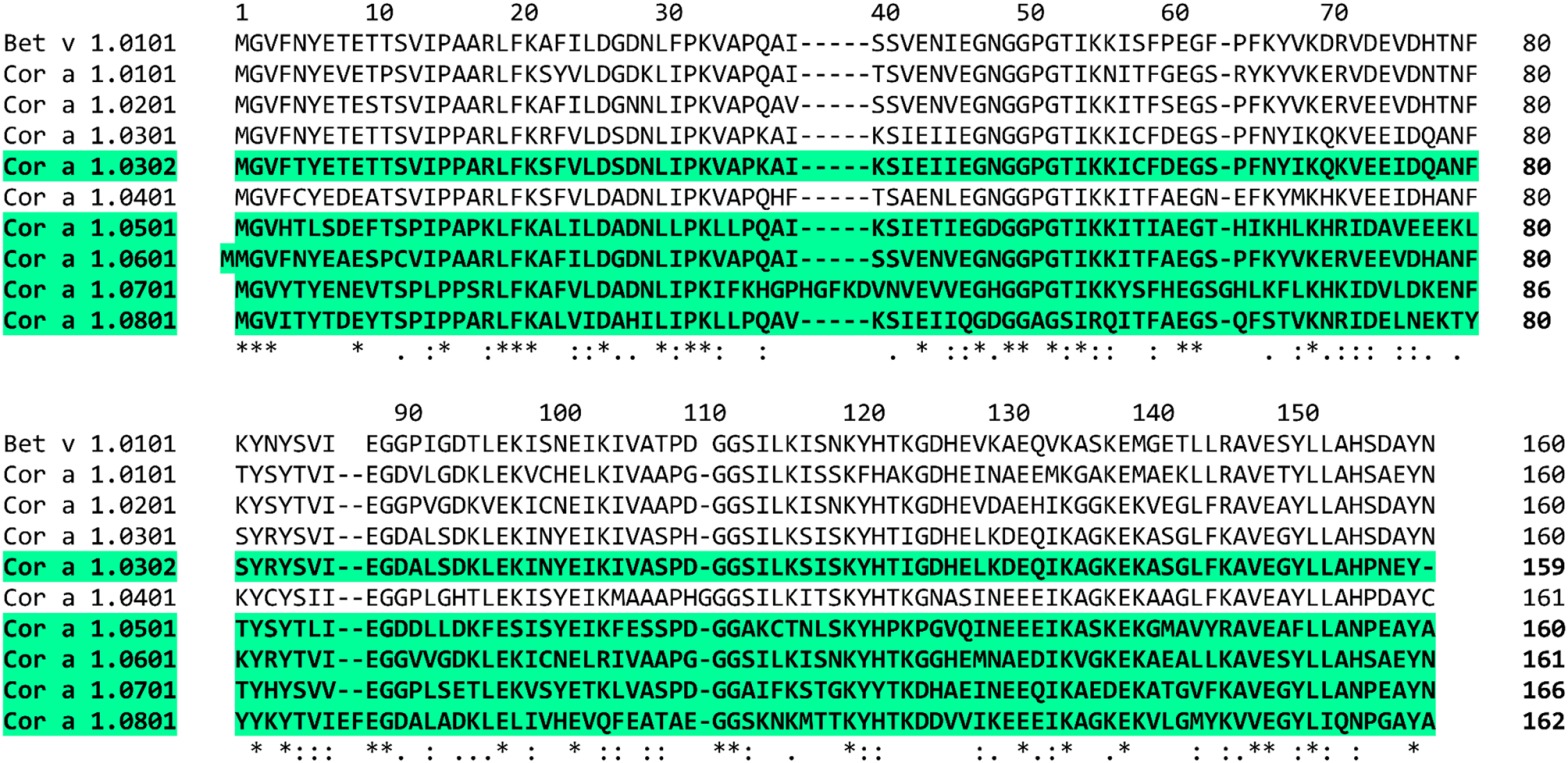
Amino acid sequence alignment of identified cor a 1 genes expressed in *Corylus avellana*. The newly identified isollargens are highlighted in green and are shown in comparison with the known Cor a 1 sequences. * conserved amino acids; : conservative exchanges; . semi-conservative exchanges.

Our data show that there exist more isoallergens and variants of Cor a 1 than previously known, e.g., four potential variants of the newly identified isoallergen Cor a 1.05 as well as three additional variants of Cor a 1.03 could be detected (Figs. 4, S1, S3). We have already shown that Cor a 1.0401 binds to the specific natural ligand Q3O-(Glc)-Gal^17^. To obtain further information on ligand binding, we are currently conducting experiments to show whether the newly identified isoallergens have different ligand binding affinities.

Since little was previously known about the expression of Cor a 1 in different plant tissues the results presented here (Fig. 1, Table 1) show for the first time, that the expression of the isoallergens studied is dependent on the plant tissue, e.g. Cor a 1.01 could be found in all tissues, whereas Cor a 1.08 was only present in the female tissues, albeit at different mRNA expression levels. Obviously, the localization of a certain isoallergen has an impact on its expression level (Fig. 1). These differences suggest different functions of the isoallergens, e.g., during plant development, fertilization and germination, that remain to be elucidated in the future.

The mRNA levels of Cor a 1.01 and Cor a 1.03 are high in pollen (Fig. 1) and thus the proteins appear to play an important role as triggers of pollen allergy. In contrast, the other isoallergens, present in mature nuts, might be relevant for allergic reactions caused after intake of hazel nut containing foodstuff (Fig. 1).

The allergenic potential of Cor a 1 proteins is likely to be enhanced by the folding stability of the isoallergens (Fig. 2). All isoallergens regained their secondary structure elements after short heating to 95 °C and recooling (Fig. 2, panel A). Similar results have already been shown for Bet v 1.0101 as well as for the homologous carrot allergen Dau c 1 (Jacob u. a. 2020; Machado u. a. 2016; Hendrich u. a. 2023). For Bet v 1.0101 it has been demonstrated that the increased fold stability of the protein has an impact on its allergenicity^36^. The Dau c 1.0105 isoallergen from carrot exhibits a pronounced heat stability which can be reduced but not completely abolished after heating of the food matrix^35,37^.

The immunoblots and ELISAs suggest that different Cor a 1 isoallergens are recognized by different pollen allergic patients. It depends on the individual immune system against which isoallergen the IgE antibodies are directed. Consequently, pollen allergic patients need to test whether it is safe to consume hazel nuts that have been baked, roasted or even untreated.

## Methods

### Identification of Cor a 1 genes in the hazel genome

Identification of Cor a 1 homologous genes was performed via BLAST (https://blast.ncbi.nlm.nih.gov/Blast.cgi) using the known isoallergen sequences of Cor a 1 on the nucleotide and protein level. At least four unknown potential isoallergens were identified in the genome of *Corylus avellana* cv Tombul (GenBank assembly accession number: GCA_901000735.2)^32^, with the following GenBank accession numbers: OQ450370 (Cor a 1.0302), OQ230635 (Cor a 1.0501), OQ450371 (Cor a 1.0601), OQ230636 (Cor a 1.0701) and OQ230637 (Cor a 1.0801). The program Clustal Omega (https://www.ebi.ac.uk/Tools/msa/clustalo) was used to create multiple sequence alignments and to calculate sequence identities.

### Primer design

Based on the *C. avellana* genome, primers were designed derived from the 5′ and 3′ untranslated regions or the 5′ and 3′ ends of the genes. Primers used are listed in Table S1.

### Expression analyses by mRNA isolation from different plant tissues and cDNA production

Immature and mature hazelnuts, pollen, hazel catkins, and female flowers were collected from hazel trees (*C. avellana*) on the Campus of the University of Bayreuth, in accordance with the Ethics Committee of the University of Bayreuth and with relevant institutional, national, and international guidelines and legislation. Female flowers were harvested early in January 2021 before the onset of the pollen flight. Pollen was shaken off from mature blooming catkins and cleaned through a fine metal sieve. The different plant materials were stored at − 80 °C. As previously described^34^, total RNA was isolated from ~ 200 mg plant material using the RNeasy Plant Mini Kit (Qiagen, Hilden, Germany), followed by reverse transcription with a poly(T) primer to obtain cDNA from mRNA using the SuperScript IV First-Strand Synthesis System with ezDNase enzyme (Thermo Fisher Scientific, Germany) and the designed primer pairs (Table S1). 18S rRNA and the specific PCR products thereof (Table S1) were obtained similarly and used as a control to determine the efficiency of RNA isolation.

### Cloning procedures, sequencing and plasmid preparation

PCR amplificates using Cor a 1 specific primers (Table S1) were loaded onto 1.5% agarose gels to visualize gene expression and purified via gel extraction (Qiaquick Gel Extraction Kit, Qiagen, Germany). In case no band could be detected on the gels, slices were cut out from the gel at the corresponding heights and also purified via gel extraction. 5 µl of the extracted sample were used as a template for a second PCR using the same conditions as described above for the first PCR. To determine whether the expected Cor a 1 gene was present in the PCR amplificate, the PCR products were either sequenced directly using the amplifcation primers and/or they were cloned into the pCR-Blunt vector using the Zero Blunt PCR Cloning Kit (Thermo Fisher Scientific Germany) as previously described^34^. Sequences were analyzed by Sanger sequencing of the isolated plasmids or by colony sequencing (Microsynth AG, Lindau, Germany). Four to ten clones were subjected to sequencing for each Cor a 1 gene detection.

The genes of Cor a 1.0501, 1.0601, 1.0701 and 1.0801 identified by sequencing of the pCRBlunt plasmids were cloned into the bacterial expression vector pET-GB1a (G. Stier, EMBL) via Gibson cloning^38^ using the primers shown in Table S2. Thus, the proteins contain an N-terminal 6His-GB1a fusion which can be cleaved off by tobacco etch virus (TEV) protease. All cleaved off Cor a 1 proteins start N-terminally with the amino acid sequence GMGV. The Cor a 1.0302 gene was ordered in the pET11a expression vector (Eurofins Genomic, Ebersberg, Germany), resulting in a tagless protein starting with a methionine.

### *LC-MS*^*E*^* analysis*

LC-MS^E^ analysis of pollen extracts was performed applying published protocols^17,34,35^. Differing from this a Uniprot database was used for MS database search. The database consisted of reviewed entries of all species as of 2022.09 and amino acid sequences translated from nucleic acid sequences mentioned above. To obtain hazelnut extract for LC-MS^E^ analysis, procedures were similar to those previously described^24^. The LC-MS^E^ analysis was performed as described above for pollen extracts. In brief, mature hazelnuts (Seeberger GmbH, Ulm, Germany) from the supermarket were crushed in a blender, ground in liquid nitrogen, and stirred overnight at 4 °C in 10 mM Na_2_HPO_4_/NaH_2_PO_4_ pH 7.0, 2% (w/v) polyvinylpolypyrrolidone, 2 mM EDTA, and 2 mM sodium azide. After centrifugation (20 min, 6000 g, 4 °C), the supernatant was passed through a paper filter (Rotilabo-folded filters, type 113P, Carl Roth GmbH, Karlsruhe, Germany). After dialysis against 5 L 10 mM Na_2_HPO_4_/NaH_2_PO_4_ pH 7.0 overnight, the protein sample was separated on a 19% SDS-PA gel. A protein band of the size of ca. 18 kDa corresponding to nCor a 1 was cut out and prepared for LC-MS^E^ as described^34^.

### Protein purification of Cor a 1 proteins recombinantly expressed in E. coli

Purification of rCor a 1.0501, 0601, 0701 and 0801 was performed as previously described^37^ omitting the size exclusion chromatography. Elution of the fusion proteins was performed on a 5 ml HisTrap HP column (Cytiva Europe GmbH, Freiburg, Germany) by a stepwise increase (15%, 25%, 50%, 70%, 100%) of the imidazole concentration.

pET11a_Cor a 1.0302 was expressed in BL21(DE3). Bacteria were grown in lysogeny broth (LB) supplemented with 100 µg/ml ampicillin at 37 °C. At an optical density at 600 nm of 0.6–0.7, the temperature was decreased to 18 °C and overexpression was induced overnight for ca. 18 h with 0.2 mM isopropyl β-D-1-thiogalactopyranoside (IPTG). After harvesting (6000 × g, 10 min, 4 °C), the cell pellet was resuspended in 20 mM TrisHCl pH 8.0, supplemented with DNase I and EDTA-free protease inhibitor and lysed as previously described^37^. The supernatant was purified by ion exchange chromatography using a 5 ml HiTrap Q XL column (Cytiva Europe GmbH) in 20 mM TrisHCl pH 8.0. rCor a 1.0302 was eluted using a stepwise increase (15%, 25%, 50%, 75%, 100%) of the elution buffer (20 mM TrisHCl, 500 mM NaCl pH 7.0). Fractions containing the Cor a 1.0302 protein were pooled. After the addition of 1 M (NH_4_)_2_SO_4_ the protein was further purified by hydrophobic interaction chromatography with a 5 ml HiTrap Octyl FF column (Cytiva Europe GmbH). After washing with 10 mM Na_2_HPO_4_/NaH_2_PO_4_ buffer containing 1 M ammonium sulfate, Cor a 1.0302 was eluted with 10 mM Na_2_HPO_4_/NaH_2_PO_4_.

### CD und NMR spectroscopy

Circular dichroism (CD) spectra and standard 1D ^1^H NMR spectra were recorded as previously described^37^.

### Patients’ Sera

Sera were obtained from patients after informed consent and approval by the Ethics Committee of the University of Bayreuth. All experiments were performed in accordance with relevant guidelines and regulations. Sera from patients diagnosed with seasonal (early spring) rhinitis, cough and breathing difficulties were tested for specific IgE against a tree pollen mixture including birch, hazel and alder pollen (Spez. IgE Kit from Gold Standard Diagnostics, Kassel, Germany). Only sera reaching values ≥ 3.5 U/ml corresponding to the enzyme-allergo-sorbent-test (EAST) class 3 or higher were selected and tested further against Bet v 1.0101 (used as a control), as well as hazel pollen extract and an extract from mature hazel nuts to confirm the presence of specific IgE antibodies against Cor a 1 proteins. Those sera were used in this study to detect new Cor a 1 isoallergens.

### IgE immunoblots and IgE ELISAs

IgE immunoblots and ELISAs were performed similarly to previously described procedures^37,39^. Mouse anti-human IgE coupled to alkaline phosphatase (BD Bioscience, Heidelberg, Germany) and AP Conjugate Substrate Kit (Bio-Rad, Germany) was used for immunoblots. Horse serum was used as a negative control and a commercially available mixture of sera from birch pollen and hazel pollen allergic patients (Gold Standard Diagnostics Kassel GmbH) as a positive control.

For ELISAs 2 µg/ml antigen (Cor a 1 proteins or Bet v 1.0101) was coated on high binding ELISA plates F (Sarstedt, Nümbrecht, Germany) in coating buffer (50 mM Na_2_CO_3_/NaHCO_3_ pH 9.6) overnight at 4 °C. After washing three times with PBS-T (phosphate buffered saline, 0.05% Tween 20) and blocking with blocking buffer (PBS-T containing 2% BSA) for 1 h at room temperature (RT), sera (diluted 1:10 with blocking buffer) were added and incubated overnight at RT. After washing four times with PBS-T, bound specific IgE was detected with horse radish peroxidase (HRP) labeled mouse-anti-human IgE-Fc-HRP (SouthernBiotech, Birmingham, USA) (diluted 1:5000 with blocking buffer). After 2 h at RT and washing with PBS-T, 100 µl substrate solution of the KPL TMB Microwell Peroxidase Substrate System (2-C) (Sera care Life Science, MA, USA) was added. The color reaction was stopped with 25% sulfuric acid (50 µl/well) and absorbance was read at 450 nm and 620 nm as a reference.

### Supplementary Information

Supplementary Information.

## Data Availability

All data generated or analysed during this study are included in this published article (and its Supplementary Information files). *Accession numbers of proteins:* Cor a 1.0302: *Acc. no. OQ450370*; Cor a 1.0501: *Acc. no. OQ230635*; Cor a 1.0601: *Acc. no. OQ450371*; Cor a 1.0701: *Acc. no. OQ230636*; Cor a 1.0801: *Acc. no. OQ230637*.
