# Supplementary material for: Allergenicity and structural properties of new Cor a 1 isoallergens from hazel identified in different plant tissues
Source: Sci Rep. 2024 Mar 7;14:5618. doi: 10.1038/s41598-024-55856-2 (PMC10920870; doi:10.1038/s41598-024-55856-2)
Supplement: Supplementary file 1 — Supplementary Information. [file 41598_2024_55856_MOESM1_ESM.pdf]

# Supplement

## Tables

### Table S1. Primers used for isoallergen identification at the mRNA level

| **Primer** | **Sequence of primer pairs** (5’ > 3’) | localization |
|---|---|---|
| Cor a 1.01 | fwd: CCTCTTCCCATATTCTTAATTCCTTAGATC<br>rev: GGGCGAAGACACAAGACGAGG | UTR |
| Cor a 1.02 | fwd: CCTCTACTACGTACTCCTCTTCATATTCTTCC<br>rev: GAAGATGATGATCACAATTCACAACCGC | UTR |
| Cor a 1.03 | fwd: CCTCTAGTCCTCATATATCTCCCAACCTC<br>rev: CCTTAACACGTGATGGCGGAAATAAGC | UTR |
| Cor a 1.03 | fwd: ATGGGTGTGTTCACTTATGAAACCG<br>rev: TTAATACTCATTAGGGTGTGCCAAGAGG | Start, stop codon |
| Cor a 1.04 | fwd: ATGGGTGTTTTCTGCTACGAGG<br>rev: CTAACAGTAGGCATCAGGGTGTG | Start, stop codon |
| Cor a 1.05 | fwd: GTTGTGCACCATTCCAATCAGTCCTCC<br>rev: GCGCCACAAAGCAAAGACATAGAACCTC | UTR |
| Cor a 1.06 | fwd: CCAACTTTGATCAATCCTACTACTCCTCG<br>rev: CATTACTGGGCGAAGACACAATATATATATATAAGAC | UTR |
| Cor a 1.07 | fwd: GGCAAGTACGTTCTCACATCTCCACAAC<br>rev: GAAGGCCTTAAGACAGTTTTGACCAGACAAATC | UTR |
| Cor a 1.07 | fwd: ATGGGTGTTTACACATATGAGAATG<br>rev: TTAGTTGTAGGCTTCAGGATTTG | Start, stop codon |
| Cor a 1.08 | fwd: GCCTAGCTAGTTTCATTAATCCTCAAGAAAGG<br>rev: CACCATCACTATTCACTAGTGATTGAGACTTATTAT | UTR |
| 18S rRNA | fwd: CTGGTCTTAATTGGCCGGGT<br>rev: CAGGCTGAGGTCTCGTTC | |

**Table S2. Primers used for cloning of Cor a 1 genes into pET-GB1a via Gibson cloning.**

| | Amplification | Sequence of primer pairs (5’ > 3’) |
|---|---|---|
| Cor a 1.0501 | Vector | fwd: CCTGAGGCCTATGCTTGAGGTACCGGATCCGAATTC<br>rev: CACTTAGAGTGTGAACACCCATGCCCTGAAAATAAAGATTCTCAG |
| | Insert | fwd: CTGAGAATCTTTATTTTCAGGGCATGGGTGTTCACACTCTAAGTG<br>rev: GAATTCGGATCCGGTACCTCAAGCATAGGCCTCAGG |
| Cor a 1.0601 | Vector | fwd: CGCATAGTGCTGAGTACAACTAAGGTACCGGATCCGAATTC<br>rev: CGTAGTTAAACACGCCCATGCCCTGAAAATAAAGATTCTCAG |
| | Insert | fwd: CTGAGAATCTTTATTTTCAGGGCATGGGCGTGTTTAACTACG<br>rev: GAATTCGGATCCGGTACCTTAGTTGTACTCAGCACTATGCG |
| Cor a 1.0701 | Vector | fwd: CAAATCCTGAAGCCTACAACTAAGGTACCGGATCCGAATTC<br>rev: CATTCTCATATGTGTAAACACCCATGCCCTGAAAATAAAGATTCTCAG |
| | Insert | fwd: CTGAGAATCTTTATTTTCAGGGCATGGGTGTTTACACATATGAGAATG<br>rev: GAATTCGGATCCGGTACCTTAGTTGTAGGCTTCAGGATTTG |
| Cor a 1.0801 | Vector | fwd: CTGGCGCCTATGCTTAAGGTACCGGATCCGAATTC<br>rev: TGTGTAAGTGATTACGCCCATGCCCTGAAAATAAAGATTCTCAG |
| | Insert | fwd: CTGAGAATCTTTATTTTCAGGGCATGGGCGTAATCACTTACACA<br>rev: GAATTCGGATCCGGTACCTTAAGCATAGGCGCCAG |

# Figures

## Figure S1

Cora1.02-Cav01g11820 (https://www.hardwoodgenomics.org/)

```
cct cta cta cgt act cct ctt cat att ctt cca ttt cca tag atc atc atc atc atc atc
 -   -   -   -   -   -   -   -   -   -   -   -   -   -   -   -   -   -   -   -
atc atc atg ggt gtt ttc aat tac gag gct gag acc acc tcc gtt att cca gca gct agg
 -   -   M   G   V   F   N   Y   E   A   E   T   T   S   V   I   P   A   A   R
ctg tct aag gcc ttt atc ctt gat ggc aat aac ctc atc cca aag gtt gca cct caa gct
 L   S   K   A   F   I   L   D   G   N   N   L   I   P   K   V   A   P   Q   A
gtt agc agt gtt gaa aac gtt gaa gga aat gga ggg cca gga aca atc aag aag atc acc
 V   S   S   V   E   N   V   E   G   N   G   G   P   G   T   I   K   K   I   T
ttt tcc gaa ggc agt cct ttc aag tac gtg aag gag agg gtt gaa gag gtt gac cac aca
 F   S   E   G   S   P   F   K   Y   V   K   E   R   V   E   E   V   D   H   T
aac ttc aaa tac agc tac acc gtc atc gag ggt ggt ccc gtg gga gac aaa gtg gag aag
 N   F   K   Y   S   Y   T   V   I   E   G   G   P   V   G   D   K   V   E   K
atc tgt aac gag ata aag att gtg gca gcc cct gat gga gga tcc atc ttg aag atc tcc
 I   C   N   E   I   K   I   V   A   A   P   D   G   G   S   I   L   K   I   S
aac aag tac cac acc aaa ggt gac cat gag gtg gat gca gag cat att aag ggt ggc aaa
 N   K   Y   H   T   K   G   D   H   E   V   D   A   E   H   I   K   G   G   K
gaa aag gtt gag ggt ctt ttc agg gcg gtt gag gcc tac ctc ttg gca cac tct gct gaa
 E   K   V   E   G   L   F   R   A   V   E   A   Y   L   L   A   H   S   A   E
tac aac taa aaa cct taa ttg tct ttt ggc ttt cat ttt gtc ttt aat ttc ggc ttg cta
 Y   N   -   -   -   -   -   -   -   -   -   -   -   -   -   -   -   -   -   -
... ... ... gcg gtt gtg aat tgt gat cat cat ctt c
 -   -   -   -   -   -   -   -   -   -   -   -
```

Cora1.0302 (GenBank accession number: OQ450370). Cav01g11830, Cav01g11840 (https://www.hardwoodgenomics.org/)

```
atg ggt gtg ttc act tat gaa acc gag acc act tca gtt atc cct ccg gct agg ctg ttc
 M   G   V   F   T   Y   E   T   E   T   T   S   V   I   P   P   A   R   L   F
aag agc ttt gtc cta gat tcc gac aac ctc atc cca aag gtt gct cca aag gcc atc aag
 K   S   F   V   L   D   S   D   N   L   I   P   K   V   A   P   K   A   I   K
agc att gaa atc atc gaa gga aat gga ggt ccc gga acc att aag aag atc tgc ttc gat
 S   I   E   I   I   E   G   N   G   G   P   G   T   I   K   K   I   C   F   D
gaa ggc agc cca ttc aac tac ata aag caa aag gtt gaa gag att gac caa gca aac ttt
 E   G   S   P   F   N   Y   I   K   Q   K   V   E   E   I   D   Q   A   N   F
tca tat cgc tac agt gtg att gaa ggc gat gct ttg tcc gac aaa ctg gag aaa atc aat
 S   Y   R   Y   S   V   I   E   G   D   A   L   S   D   K   L   E   K   I   N
tac gag atc aag ata gtg gca tcc cct gat gga gga tct atc ttg aag agc atc agc aag
 Y   E   I   K   I   V   A   S   P   D   G   G   S   I   L   K   S   I   S   K
tac cac acc ata gga gac cat gaa ctc aag gac gag cag att aag gct gga aaa gag aag
 Y   H   T   I   G   D   H   E   L   K   D   E   Q   I   K   A   G   K   E   K
gcc tca gga ctt ttc aaa gct gtt gag ggc tac ctc ttg gca cac cct aat gag tat taa
 A   S   G   L   F   K   A   V   E   G   Y   L   L   A   H   P   N   E   Y   -
```

Cora1.0501: (GenBank accession number: OQ230635)

```
ttg tgc acc att cca atc agt cct cca ttt tct acc att tct aaa ttc tga ata tat tcg
 -   -   -   -   -   -   -   -   -   -   -   -   -   -   -   -   -   -   -   -
atc atg ggt gtt cac act cta agt gat gag ttc act agc ccc atc cca gca cca aag ctg
 -   M   G   V   H   T   L   S   D   E   F   T   S   P   I   P   A   P   K   L
ttc aag gcc ttg atc ctt gat gct gac aac ctc ctt ccc aag ctc ctg cct cag gcc att
 F   K   A   L   I   L   D   A   D   N   L   L   P   K   L   L   P   Q   A   I
aag agt atc gag acg att gag ggc gat gga ggg cct gga acc atc aag aag atc act atc
 K   S   I   E   T   I   E   G   D   G   G   P   G   T   I   K   K   I   T   I
gct gaa ggt acc cat atc aag cac ttg aag cat agg att gat gca gta gag gaa gag aaa
 A   E   G   T   H   I   K   H   L   K   H   R   I   D   A   V   E   E   E   K
```

**ttg aca tac agt tac aca ctg att gag ggt gat gat ttg ctg gac aag ttt gaa tca att**
**L T Y S Y T L I E G D D L L D K F E S I**
**tct tat gag att aag ttt gag tcc tct cct gat ggg gga gcc aaa tgt aca aat ctt agc**
**S Y E I K F E S S P D G G A K C T N L S**
**aag tac cat cct aaa cca ggg gtc cag atc aat gaa gag gaa atc aag gca agc aag gaa**
**K Y H P K P G V Q I N E E E I K A S K E**
**aag ggc atg gct gtt tac aga gcc gtg gaa gcc ttc ctc ttg gcc aat cct gag gcc tat**
**K G M A V Y R A V E A F L L A N P E A Y**
**gct tga** tgc ttc ctt gtt gag aca cta tta tat gta tct cgt gtt cct caa agt tta ttg
**A -** - - - - - - - - - - - - - - - - - -
agg ttc tat gtc ttt gct ttg tgg cgc
- - - - - - - - -

Cora1.0601: (GenBank accession number: OQ450371)

aac ttt gat caa tcc tac tac tcc tcg atc atc atc atc atc atc atc **atg atg ggt gtt**
- - - - - - - - - - - - - - - - **M M G V**
**ttc aat tac gag gct gag agc ccc tgt gtt atc cca gca gct agg ctg ttc aag gcc ttt**
**F N Y E A E S P C V I P A A R L F K A F**
**atc ctt gat ggc gat aac ctc atc cca aag gtt gca cct caa gct att agc agc gtt gaa**
**I L D G D N L I P K V A P Q A I S S V E**
**aac gtt gaa gga aat gga ggg ccc gga acc atc aag aag atc acc ttt gcc gaa ggc agc**
**N V E G N G G P G T I K K I T F A E G S**
**cct ttc aag tac gtg aag gag agg gtt gag gag gtt gac cac gca aac ttc aaa tac aga**
**P F K Y V K E R V E E V D H A N F K Y R**
**tac acc gtg atc gag ggc ggt gtc gtg ggc gat aag ctg gag aag atc tgc aac gag ctg**
**Y T V I E G G V V G D K L E K I C N E L**
**agg ata gtg gca gcc cct ggt ggt gga tcc atc ttg aag atc agc aac aag tac cac acc**
**R I V A A P G G G S I L K I S N K Y H T**
**aaa ggc ggc cat gag atg aat gca gag gat att aag gtt ggc aaa gaa aag gcc gag gca**
**K G G H E M N A E D I K V G K E K A E A**
**ctt tta aag gca gtt gag agc tac ctc ttg gca cac tct gct gaa tac aac taa** acc tta
**L L K A V E S Y L L A H S A E Y N -** - -
att gtc tta tat ata tat att gtg tct tcg ccc agt aat
- - - - - - - - - - - - -

Cora1.0701: (GenBank accession number: OQ230636)

gca agt acg ttc tca cat ctc cac aac ... ... ... ctt ctt ctt cct ttg atc atc ttc
- - - - - - - - - - - - - - - - - - - -
cta gtc ttc cta gat atc atc **atg ggt gtt tac aca tat gag aat gag gtc acc tcc ccc**
- - - - - - - **M G V Y T Y E N E V T S P**
**ctc cct cca tcg agg ttg ttc aag gcc ttc gtc ctt gat gcc gac aac ctt atc ccc aag**
**L P P S R L F K A F V L D A D N L I P K**
**att ttc aag cat gga ccg cac ggt ttt aag gat gtg aac gtg gaa gtc gtt gaa ggc cat**
**I F K H G P H G F K D V N V E V V E G H**
**gga ggg cca gga acc atc aaa aag tat tcg ttt cat gaa ggc agc ggg cac ttg aaa ttt**
**G G P G T I K K Y S F H E G S G H L K F**
**ttg aag cac aag att gat gtg cta gac aag gaa aac ttc aca tac cat tac agc gtg gtt**
**L K H K I D V L D K E N F T Y H Y S V V**
**gaa ggt ggt ccg ttg tcg gag aca ctc gag aaa gtc tca tac gag acc aag ttg gtg gct**
**E G G P L S E T L E K V S Y E T K L V A**
**tcc cct gac gga gga gcc atc ttc aag agc acc ggc aag tat tat aca aaa gat cac gct**
**S P D G G A I F K S T G K Y Y T K D H A**
**gag atc aat gaa gag caa atc aag gct gaa gat gaa aag gcc acg ggg gtg ttc aag gct**
**E I N E E Q I K A E D E K A T G V F K A**
**gtt gaa ggc tac ctc ttg gca aat cct gaa gcc tac aac taa agc aag ttg cta aat tta**
**V E G Y L L A N P E A Y N - - - - - - -**
atg ttg cat gtg tac gta att gta tct cat cga ttt gtc tgg tca aaa ctg tct taa ggc
- - - - - - - - - - - - - - - - - - - -
ctt c
-

Cora1.0801: (GenBank accession number: OQ230637). Cav01g11530 (https://www.hardwoodgenomics.org/)

```
cct agc tag ttt cat taa tcc tca aga aag gat atc atg ggc gta atc act tac aca gat
 -   -   -   -   -   -   -   -   -   -   -   -   M   G   V   I   T   Y   T   D
gag tac acc tct cct atc cca cca gct aga ttg ttc aaa gcc ttg gtc att gat gct cac
 E   Y   T   S   P   I   P   P   A   R   L   F   K   A   L   V   I   D   A   H
atc ctc atc cca aaa ctc ctc cca cag gct gtt aag agc att gaa atc att caa ggc gat
 I   L   I   P   K   L   L   P   Q   A   V   K   S   I   E   I   I   Q   G   D
gga ggg gct gga agc atc agg caa atc act ttt gcc gaa ggt agc caa ttt agt act gtc
 G   G   A   G   S   I   R   Q   I   T   F   A   E   G   S   Q   F   S   T   V
aag aac cga att gat gag ctg aat gaa aaa act tat tac tac aag tac acg gtg atc gaa
 K   N   R   I   D   E   L   N   E   K   T   Y   Y   Y   K   Y   T   V   I   E
ttc gaa ggt gat gcc ttg gct gac aag ctt gaa ttg att gtt cat gag gtt caa ttt gag
 F   E   G   D   A   L   A   D   K   L   E   L   I   V   H   E   V   Q   F   E
gca acg gct gaa ggt ggg agt aaa aat aag atg aca acc aag tac cat acc aag gat gac
 A   T   A   E   G   G   S   K   N   K   M   T   T   K   Y   H   T   K   D   D
gta gtg atc aag gaa gag gaa atc aag gct ggc aag gaa aag gtg ctg ggt atg tac aaa
 V   V   I   K   E   E   E   I   K   A   G   K   E   K   V   L   G   M   Y   K
gtt gtg gaa ggc tac ctc atc cag aac cct ggc gcc tat gct taa tta atg cta atc tcc
 V   V   E   G   Y   L   I   Q   N   P   G   A   Y   A   -   -   -   -   -   -
aat cag tct ctt ttg tct act gtt taa tgt tgt ggc ttt tac ctt tgt ctt tga ata atc
 -   -   -   -   -   -   -   -   -   -   -   -   -   -   -   -   -   -   -   -
gcc tta atg tgg tgt gat aat aag tct caa tca cta gtg aat agt gat ggt g
 -   -   -   -   -   -   -   -   -   -   -   -   -   -   -   -
```

**Figure S1: Coding sequences of Cor a 1 isoallergens and flanking regions used for the selection of PCR primers.** The coding sequences and the corresponding amino acid sequences are depicted in bold letters. Underlined sequences represent the primer sequences used for PCR amplification. They are based on the genome sequences of the Turkish cultivar “Tombul” (Lucas u. a. 2021). Dotted sections indicate regions that were not covered by sequencing.

# Figure S2

**Hazel pollen extract:**

```
                  1                  19  22 23       30   34   38  41              56 57          65 67  70
Cor a 1.0101      MGVFNYEVETPSVIPAAR LFK S--YVLDGDK LIPK VAPQ-AIT-SVENVEGNGGPGTIK N--ITFGEGSR YK YVK ER
Cor a 1.0104      MGVFNYEVETPSVISAAR LFK S--YVLDGDK LIPK VAPQ-AIT-SVENVGGNGGPGTIK N--ITFGEGSR YK YVK ER
Cor a 1.0301      MGVFNYETETTSVIPPAR LFK R  FVLDSDN-LIPK VAPK AIK SIEIIEGNGGPGTIK K  ICFDEGSP-FN-YIK QK
Cor a 1.0302      MGVFTYETETTSVIPPAR LFK S--FVLDSDN-LIPK VAPK AIK SIEIIEGNGGPGTIK K  ICFDEGSP-FN-YIK QK

                  72          82 84             96  99     105            117  121  125     131 133 136 139 141 144 147
Cor a 1.0101      VDEVDNTNFT-YS-YTVIEGDVLGDK LEK VCHELK IVAAPGGGSILK ISSK FHAK GDHEIN-AE--EMK GAK EM--AEK LLR AVETYLLAHSAEYN
Cor a 1.0104      VDEVDNTNFK YS-YTVIEGDVLGDK LEK VCSELK IVAAPGGGSTLK ISSK FHAK GDHEIN-AE--EMK GAK EM--AEK LLR AVETYLLAHSAEYN
Cor a 1.0301      VEEIDQANFS-YR YSVIEGDALSDK LEK INYEIK IVASPHGGSILK SISK YHTI-GDHELK DE--QIK AGK EK  ASG-LFK AVEGYLLAHSDAYN
Cor a 1.0302      VEEIDQANFS-YR YSVIEGDALSDK LEK INYEIK IVASPDGGSILK SISK YHTI-GDHELK DE--QIK AGK EK  ASG-LFK AVEGYLLAHPNEY
```

**Mature hazel nut (in-gel digestion):**

```
                  1                  19  22           34                     56 57          67  70
Cor a 1.0403      MGVFCYEDEATSVIPPAR LFK SFVLDADNLIPK VAPQHFTGAENLEGNGGPGTIK K  ITFAEGSEFK YMK HK

                  72         82                 99     105           118  122  126          137 140 142     148
Cor a 1.0403      VEEIDHANFK YCYSIIEGGPLGHTLEK ISYEIK MAAAPHGGGSILK ITSK YHTK GNASISEEEIK AGK EK  AAG-LFK AVEAYLLAHPDTYC
```

**Figure S2: Amino acid sequences of Cor a 1 peptides identified in extracts of hazel pollen and mature hazel nut by LC-MS$^{E}$**. Corresponding peptides detected by LC-MS$^{E}$ are underlined. Differing sequence stretches are color coded. Identical colors indicate identical amino acid sequences of peptides, black stretches indicate peptides which were not detected. Underlined stretches indicate unambiguously confirmed peptides. A dashed underline indicates a C-terminal peptide which is indistinguishable due to a possible deamidation N→D. The positions of the amino acids in the sequence were labeled according to the following scheme: e.g., T19 means that the corresponding peptide is released after "T"-(Trypsin) cleavage and starts at amino acid position 19.

# Figure S3

## Cor a 1.01

<u>Cor a 1.01- Cav01g11790 (flower):</u>

CCTCTTCCCATATTCTTAATTCCTTAGATCATCATCATCATGGGTGTTTTCAATTACGAGGTCGAGACCCCCTCCGTT
ATCCCAGCGGCAAGGCTGTTCAAGTCCTATGTCCTTGATGGCGATAAGCTCATCCCAAAGGTTGCACCTCAAGCT
ATTACCAGCGTTGAAAACGTTGGAGGAAATGGAGGGCCTGGAACCATCAAGAATATCACCTTTGGCGAAGGCA
GCCGTTACAAGTACGTGAAGGAGAGGGTTGATGAGGTTGACAACACAAACTTCAAATATAGCTACACCGTGATC
GAGGGTGATGTCCTGGGTGACAAGCTGGAGAAGGTCTGCAGCGAGCTGAAGATAGTGGCAGCCCCTGGTGGG
GGATCCATCTTGAAGATCAGCAGCAAGTTCCACGCCAAAGGCGACCATGAGATTAATGCAGAGGAGATGAAGG
GTGCCAAAGAAATGGCCGAGAAACTTTTAAGGGCGGTTGAGACCTACCTATTGGCACACTCTGCTGAATACAAC
TAAATATCGTCTTGTGTCTTCGCCC

<u>Cor a 1.01-like variant 1 (GenBank accession number: OQ569792) (pollen):</u>

CCTCTTCCCATATTCTTAATTCCTTAGATCATCATCATCATGGGTGTTTTCAATTACGAGGCTGAGACCACCTCTGTT
ATCCCTGCGGCAAGGCTGTTCAAGTCCTATGTCCTTGATGGCGATAAGCTCATCCCAAAGGTTGCACCTCAAGCTA
TTACCAGCGTTGAAAACGTTGGAGGAAATGGAGGGCCTGGAACCATCAAGAATATCACCTTTGGCGAAGGCAG
CCGTTACAAGTACGTGAAGGAGAGGGTTGATGAGGTTGACAACACAAACTTCACATACAGCTACACCGTGATCG
AGGGTGATGTCCTGGGTGACAAGCTGGAGAAGGTCTGCCACGAGCTGAAGATAGTGGCAGCCCCTGGTGGAG
GATCCATCTTGAAGATCAGCAGCAAGTTCCACGCCAAAGGTGACCATGAGATTAATGCAGAGGAGATGAAGGGT
GCCAAAGAAATGGCCGAGAAACTTTTAAGGGCGGTTGAGACCTACCTATTGGCACACTCTGCTGAATACAACTA
AACCTCGTCTTGTGTCTTCGCCC

<u>Cor a 1.01-like variant 2 (GenBank accession number: OR326622) (mature nut):</u>

CCTCTTCCCATATTCTTAATTCCTTAGATCATCATCATCATGGGTGTTTTCAATTACGAGGCTGAGACCACCTCTGTT
ATCCCTGCGGCAAGGCTGTTCAAGTCCTATGTCCTTGATGGCGATAAGCTCATCCCAAAGGTTGCACCTCAAGCTA
TTACCAGCGTTGAAAACGTTGGAGGAAATGGAGGGCCTGGAACCATCAAGAATATCACCTTTGGCGAAGGCAG
CCGTTACAAGTACGTGAAGGAGAGGGTTGATGAGGTTGACAACACAAACTTCACATACAGCTACACCGTGATCG
AGGGTGATGTCCTGGGTGACAAGCTGGAGAAGGTCTGCCACGAGCTGAAGATAGTGGCAGCCCCTGGTGGAG
GATCCATCTTGAAGATCAGCAGCAAGTTCCACGCCAAAGGTGACCATGAGATTAATGCAGAGGAGATGAAGGGT
GCCAAAGAAATGGCCGAGAAACTTTTAAGGGCGGTTGAGACCTACCTATTGGCACACTCTGCTGAATACAACTA
AACCTCGTCTTGTGTCTTCGCCC

## Cor a 1.02

<u>Cor a 1.02-like variant (GenBank accession number: OQ569793) (flower):</u>

ATGGGTGTTTTCAATTACGAGGCTGAGACCACCTCCGTTATTCCAGCAGCTAGGCTGTTTAAGGCCTTTATCCTT
GATGGCAATAACCTCATCCCAAAGGTTGCACCTCAAGCTGTTAGCAGTGTTGAAAACGTTGAAGGAAATGGAG
GGCCAGGAACAATCAAGAAGATCACCTTTTCCGAAGGCAGTCCTTTCAAGTACGTGAAGGAGAGGGTTGAAG
AGGTTGACCACACAAACTTCAAATACAGCTACACCGTCATCGAGGGTGGTCCCGTGGGAGACAAAGTGGAGA
AGATCTGTAACGAGATAAAGATTGTGGCAGCCCCTGATGGAGGATCCATCTTGAAGATCTCGAACAAGTACCA
CACCAAAGGTGACCATGAGGTGGATGCAGAGCATATTAAGGGTGGCAAAGAAAAGGTCGAGGGTCTTTTCAG
GGCGGTTGAGGCCTACCTCTTGGCACACTCTGCTGAATACAACTAA

## Cor a 1.03

Cor a 1.03-Cav01g11870 (immature nut):

CTCTAGTCCTCATATATCTCCCAACCTCAAAAACTCTCCACTTAATTATTATCATTCTCTCTCACATCACTTCCAAA
GTCTTAGCTCATCATGGGTGTGTTCACTTATGAAACCGAGACCACTTCAGTTATCCCTCCCGCTAGGCTGTTCAA
GAGCTTTGTCCTAGATTCCGACAACCTCATCCCAGAGGTTGCTCCAAAGGCCATCAAGAGCATTGAAATCATCG
AAGGAAATGGAGGTCCCGGAACCATTAAGAAGATCTGCTTCGATGAAGGCAGCCCAATCAGCTACATAAAGC
AAAAGGTTGATGAGATTGACCAAGCAAACTTTTCATATCGCTACAGTGTGATTGATGGCGATGCTTTGTCCGAC
AAACTGGAGAAAATCAATTGCGAGATCAAGATAGTGGCATCCCCTGATGGAGGATCTATCATGAAGAGCATCA
GCAAGGACCACACCATAGGAGACCATGAGCTCGAGGAAGAGCAGATTAAGGCTGGAAAAGAGATGGCCTCA
GGACTTTTCAAAGCTGTTGAGGACTACCTCTTGGCACACCCTAATGAGTATTAATTAAGGCTAATTGTGTGGTG
GTTATATTAATTTGCTTATTTCCGCCATCACGTGTTAAGG

Cor a 1.03-like variant 1 (GenBank accession number: OQ569794) (immature nut):

CCCAACCTCAAAAACTCTCCACTTAATTATTATCATTCTCTCTCACATCACTTCCAAAGTCTTAGCTCATCATGGG
TGTGTTCACTTATGAAACCGAGACCACTTCAGTTATCCCTCCCGCTAGGCTGTTCAAGAGCTTTGTCCTAGATTC
CGACAACCTCATCCCAGAGGTTGCTCCAAAGGCCATCAAGAGCATTGAAATCATCGAAGGAAATGGAGGTCCC
GGAACCATTAAGAAGATCTGCTTCGATGAAGGCAGCCCAATCAGCTACATAAAGCAAAAGGTTGATGAGATTG
ACCAAGCAAACTTTTCATATCGCTACAGTGTGATTGATGGCGATGCTTTGTCCGACAAACTGGAGAAAATCAAT
TGCGAGATCAAGATAGTGGCATCCCCTGATGGAGGATCTATCATGAAGAGCATCAGCAAGGACCACACCATA
GGAGACCATGAGCTCGAGGAAGAGCAGATTAAGGCTGGAAAAGAGATGGCCTCAGGACTTTTCAAAGCTGTT
GGGGACTACCTCTTGGCACACCCTAATGAGTATTAATTAAGGCTAATTGTGTGGTGGTTATATTAATTTGCTTA
TTTCCGCCATCACGTGTTAAGG

Cor a 1.03-like variant 2 (GenBank accession number: OR326623) (catkin):

ATGGGTGTGTTCACTTATGAAACCGAGACCACTTCAGTTATCCCTCCGGCTAGGCTGTTCAAGAGCTTTGTCCT
AGATTCCGACAACCTCATCCCAAAGGTTGCTCCAAAGGCCATCAAGAGCATTGAAATCATCGAAGGAAATGGA
GGTCCCGGAACCATTAAGAAGATCTGCTTCGATGAAGGCAGCCCATTCAACTACATAAAGCAAAAGGTTGAAG
AGATTGACCAAGCAAACTTTTCATATCGCTACAGTGTGATTGAAGGCGATGCTTTGTCCGACAAACTGGAGAA
AATCAATTACGAGATCAAGATAGTGGCATCCCCTGATGGAGGATCTATCATGAAGAGCATCAGCAAGGACCAC
ACCATAGGAGACCATGAGCTCAAGGAAGAGCAGATTAAGGCTGGAAAAGAGATGGCCTCAGGACTTTTCAAA
GCTGTTGAGGACTACCTCTTGGCACACCCTAATGAGTATTAA

## Cor a 1.04

Cor a 1.04-like variant 1 (GenBank accession number: OQ569795) (immature nut):

CCTCTACTACGTACTCCTCTTCATATTCTTCCATTTCCATAGATCATCATCATCATCATCATCATCATGGGTGTTTT
CTGCTACGAGACTGAGACCACCTCCGTTATCCCTCCGGCTAGGCTGTTCAAGTCCTTTGTCCTAGATGCCGACA
ACCTCATTCCCAAGGTTGCTCCTCAGCACTTCACCGGCGCTGAAAACCTCGAAGGAAATGGCGGGCCTGGAAC
CATCAAGAAGATCACCTTCGCCGAAGGCAGCGAATTCAAGTACATGAAGCACAAGGTTGAGGAGATCGACCA
CGCAAACTTCAAATACTGCTACAGCATCATCGAGGGAGGTCCATTGGGGCACACACTGGAGAAGATCTCTTAC
GAGATCAAGATGGCGGCAGCCCCTCATGGAGGAGGATCCATCTTGAAGATCACCAGCAAGTACCACACCAAG
GGCAACGCTTCAATCAGTGAGGAGGAGATCAAGGCTGGCAAAGAGAAGGCCGCCGGACTTTTCAAGGCTGTT
GAGGCTTACCTCTTGGCACACCCTGATACCTACTGTTAGAGTAGCTAACACCCACGTACAACTAAAGCTTGTGT
TGTGTTGTGTGGTCCCTCAATAATGAGTTGTTACTGCTTGATTTTGGTTTGCTAATAAAGGAGTTTGCGGTTGT
GAATTGTGATCATCATCTTC

## Cor a 1.05

Cor a 1.05-like variant 1 (GenBank accession number: OQ569796) (catkin):

GTTGTGCACCATTCCAATCAGTCCTGCATTTTCTACCATCTCTAAATTCTGAATATATTCGATCATGGGTGTTCAC
ACTCTAAGTGATGAGGTCACTAGCCCCATCCCAGCACCAAAGCTGTTCAAGGCCTTGATCCTTGATGCTGACAA
CCTCCTTCCCAAGCTCCTGCCTCAGGCCATTAAGAGTATCGAGACAGTTGAGGACAATGGAGGGCCTGGAACC
ATCAAGAAGATCACCATCGCTGAAGGTACCCACATCAAGCACTTGAAGCATAGGATTGATGCAGTAGACGAAG
AGAAATTGACATACAGTTACACACTGATTGAGGGTGATGATTTGCTGGACAAGTTTGAATCAATTTCTTATGAG
ATTAAGTTTGAGTCTTCTCCTGATGGGGGAGCCAAATGTACAAATTTTAGCAAGTACCATCCTAAACCAGGGGC
CCAGATCAATGAAGAGGAAATGAAGGCAAGCAAGGAAAAGGGCTTGGCTGTTTACAGAGCTATGGAAGCCTA
CCTCTTGGCCAATCCTGAAGCCTATGCTTGATGTTTCCTTATTGAGACACTATATATGTATCCTGTGTTCCTCAAA
GTGTATTGAGGTTCTATGTCTTTGCTTTGTGGCGC

Cor a 1.05-like variant 2 (GenBank accession number: OR232687) (mature nut)

CTAATTCTGATATATTCGATCATGGGTGTTCACACTCTAAGTGATGAGTTCACTAGCCCCATCCCAGCACCAAA
GCTGTTCAAGGCCTTGATCCTTGATGCTGACAACCTCCTTCCCAAGCTCCTGCCTCAGGCCATTAAGAGTATCG
AGACGGTTGAGGGCGATGGAGGGCCTGGAACCATCAAGAAGATCACTATCGCTGAAGGTACCCATATCAAGC
ACTTGAAGCATAGGATTGATGCAGTAGAGGAAGAGAAATTGACATACAGTTACACACTGATTGATGGTGATG
ATTTGCTGGACAAGTTTGAATCAATTTCTTATGAGATTAAGTTTGAGTCCTCTCCTGATGGGGGAGCCAAATGT
ACAAATCTTAGCAAGTACCATCCTAAACCAGGGGTCCAGATCAATGAAGAGGAAATCAAGGCAAGCAAGGAA
AAGGGCATGGCTGTTTACAGAGCCGTGGAAGCCTTCCTCTTGGCCAATCCTGAGGCCTATGCTTGATGCTTCCT
TGTTGAGACACTATTATATGTATCTCGTGTTCCTCAAAGTTTATTGAGGTTCTATGTCTTTGCTTKGTGGCGCAA
AA

Cor a 1.05-Cav01g11940 (flower):

GTTGTGCACCATTCCAATCAGTCCTCCATTTTCTACCATTTTCTAAATTCTGAATATATTCGATCATGGGTGTTCA
CACTCTAAGTGATGAGTTCACTAGCCCCATCCCAGCACCAAAGCTGTTCAAGGCCTTGATCCTTGATGCTGACA
ACCTCCTTCCCAAGCTCCTGCCTCAGGCCATTAAGAGTATCGAGACGGTTGAGGGCGATGGAGGGCCTGGAAC
CATCAAGAAGATCACTATCGCTGAAGGTACCCACATCAAGCACTTGAAGCATAGGATTGATGCAGTAGAGGAA
GAGAAATTGACATACAGTTACACACTGATTGAGGGTGATGATTTGCTGGACAAGTTTGAATCAATTTCTTATGA
GATTAAGTTTGAGTCCTCTCCTGATGGGGGAGCCAAATGTACAAATCTTAGCAAGTACCATCCTAAACCAGGG
GTCCAGATCAATGAAGAGGAAATCAAGGCAAGCAAGGAAAAGGGCATGGCTGTTTACAGAGCCGTGGAAGC
CTTCCTCTTGGCCAATCCTGAGGCCTATGCTTGATGCTTCCTTGTTGAGACACTATTATATATATCTCGTGTTCCT
CAAAGTTTATTGAGGTTCTATGTCTTTGCTTTGTGGCGC

Cor a 1.05-Cav01g11520 (immature nut):

AAATTTCCCTCTCTAATTCTGAATATATTCGATCATGGGTGTTCACACTCTAAGTGATGAGGTCACTAGCCCCAT
CCCAGCACCAAAGCTGTTCAAGGCCTTGATCCTTGATGCTGACAACCTCCTTCCCAAGCTCCTGCCTCAGGCCAT
TAAGAGTATCGAGACAGTTGAGGGCAATGGAGGGCCTGGAACCATCAAGAAGATCACCATCGCTGAAGGTAC
CCACATCAAGCACTTGAAGCATAGGATTGATGCAGTAGACGAAGAGAAATTGACATACAGTTACACACTGATT
GAGGGTGATGATTTGCTGGACAAGTTTGAATCAATTTCTTATGAGATTAAGTTTGAGTCTTCTCCTGATGGGGG
AGCCAAATGTACAAATTTTAGCAAGTACCATCCTAAACCAGGGGCCCAGATCAATGAAGAGGAAATGAAGGC
AAGCAAGGAAAAGGGCTTGGCTGTTTACAGAGCTATGGAAGCCTACCTCTTGGCCAATCCTGAAGCCTATGCT
TGATGTTTCCTTATTGAGACACTATCTATGTATCCTGTGTTCCTCAAAGTGTATTGAGGTTCTATGTCTTTGCTTT
GTGGCGC

## Cor a 1.07

Cor a 1.07-like variant 1 (GenBank accession number: OR232688) (catkin)

TCCTCTTCTTCTTCTTCTTCTTCCTAGTCTTCATAGCTAGATATCATCATGGGTGTTTTCACATATGAGAATGAGG
TCACCTCCCCCCTCCCTCCATCGAAGTTGTTCAAGGCCTTCGTCCTTGATGCCGACAACCTTGTCCCCAAGATTT
ACAAGCACGGCATAAATGACCTGAACGTGGAAATCCTTGAAGGCCATGGAGGGCCTGGAACCATCAAAAAGT
TTACGTTTCATGAAGGCGGCCACTTGAAATTTTTGAAGCACAAGGTTGATGTGCTAGACAAGGAACACTTCAC
ATACAATTACAGCGTGGTTGAAGGTGGTCCTTTGTCGGAGACACTCGAGAAAGTCTCATTCGAGACCAAGTTG
GTGGCTTCCCCAGATGGAGGAACCATCTTCAAGAGCACCGGCAAGTATTATACAAAAGATCACGCTGAGATAA
ATGAAGAGAAAATCAAGGCTGAAGATGAAAAGGCCACGGGGGTGTTCAAGGCAGTTGAAGGCTACCTCTTG
GCAAATCCTGAAGCCTACAACTAAAGCTGCTTGATTGTTGCATGTATATCTGATTTGTCTGGTCAAAACTGTCTT
AAGGCCTTC

Cor a 1.07-like variant 2 (GenBank accession number: OR232689) (pollen)

TCTCTTCTTCTTCTTCCTTTTGATCATCTTCCTAGTACGCCTAGATATCATCATGGGTGTTTTCACATATGAGAAT
GAGGTCACCTCCCCCCTCCCTCCATCGAAGTTGTTCAAGGCCTTCGTCCTTGATGCCGACAACCTTGTCCCCAAG
ATTTACAAGCACGGCATAAATGACCTGAACGTGGAAATCCTTGAAGGCCATGGAGGGCCTGGAACCATCAAA
AAGTTTACGTTTCATGAAGGCGGCCACTTGAAATTCTTGAAGCACAAGGTTGATGTGCTAGACAAGGAACACT
TCACATACAATTACAGCGTGGTTGAAGGTGGTCCTTTGTCGGAGACACTCGAGAAAGTCTCATACGAGACCAA
GTTGGTGGCTTCCCCAGACGGAGGAACCATCTTCAAGAGCACCGGCAAGTATTATACAAAAGATCACGCTGAG
ATCAATGAAGAGCAAATCAAGGCTGAAGATGTAAAGGCCACAGGGGTGTTCAAGGCAGTTGAAGGTTACCTC
TTGGCAAATCCTGAAGCCTACAACTAAAGCTGCTTGATTGTTGCATGTGTATCTGATTTGTCTGGTCAAAACTGT
CTTAA

## Cor a 1.08

Cor a 1.08-like variant 1 (GenBank accession number: OR232690) (flower)

GCCTAGCTAGTTTCATTAATCCTCAAGAAAGGATATCATGGGCGTAATCACTTACGTAGACGAGTACACCTCCC
CTATTCCACCAGCCAGATTGTTCAAAGCCTTGGTCATTGATGCTCACATCCTCATCCCAAAACTCCTCCCACAGG
CTGTTAAGAGCATTGAAATCATTCAAGGCGATGGAGGGGCTGGAAGCATCAGGCAAACCACTTTTGCCGAAG
GTAGCCAATTTAGTACTGTCAAGAACCGAATTGATGAGCTGAATGAAAAAACTTATTACTACAAGTACACGGT
GATCGAATTCGAAGGTGATGCCTTGGCTGACAAGCTTGAATTGATTGTTCATGAGGTTCAATTTGAGGCAACG
GCTGAAGGTGGGAGTAAAAATAAGATGACAACCAAGTACCATACCAAGGATGACGTAGTGATCAAGGAAGA
GGAAATCAAGGCTGGCAAGGAAAAGGTGCTGGGTATGTACAAAGTTGTGGAAGGCTACCTCATCCAGAACCC
TGGCGCCTATGCTTAATTAATGCTAATCTCCAATCAGTCTCTTTTGTCTACTGTTTAATGTTGTGGCTTTTACCTT
TGTCTTTGAATAATCGCCTTAATGTGGTGTGATAATAAGTCTCAATCACTAGTGAATAGTGATGGTG

Cor a 1.08-like variant 2 (GenBank accession number: OR232691) (mature nut)

GCCTAGCTAGTTTCATTAATCCTCAAGAAAGGATATCATGGGCGTAATCACTTACACAGATGAGTACACCTCTC
CTATCCCACCAGCTAGATTGTTCAAAGCCTTGGTCATTGATGCTCACATCCTCATCCCAAAACTCCTCCCACAGG
CTGTTAAGAGCATTGAAATCATTCAAGGCGATGGAGGGGCTGGAAGCATCAGGCAAACCACTTTTGCCGAAG
GTAGCCAATTTAGTACTGTCAAGAACCGAATTGATGAGCTGAATGAAAAAACTTATTACTACAAGTACACGGT
GATCGAATTCGAAGGTGATGCCTTGGCTGACAAGCTTGAATTGATTGTTCATGAGGTTCAATTTGAGGCAACG
GCTGAAGGTGGGAGTAAAAATAAGATGACAACCAAGTACCATACCAAGGATGACGTAGTGATCAAGGAAGA
GGAAATCAAGGCTGGCAAGGAAAAGGTGCTGGGTATGTACAAAGTTGTGGAAGGCTACCTCATCCAGAACCC
TGGCGCCTATGCTTAATTAATGCTAATCTCCAATCAGTCTCTTTTGTCTACTGTTTAATGTTGTGGCTTTTACCTT
TGTCTTTGAATAATCGCCTTAATGTGGTGTGATAATAAGTCTCAATCACTAGTGAATAGTGATGGTG

**Figure S3: Sequences of additional putative Cor a 1 variants identified at the mRNA level in different hazel tissues.** Sequences labeled with a Cav01 number are present in the "Tombul"

cultivar (Lucas et al. 2021). Variants labeled with a Genbank accession number are genes identified by us which were not found in the “Tombul cultivar”. Grey: coding sequence.

## Figure S4

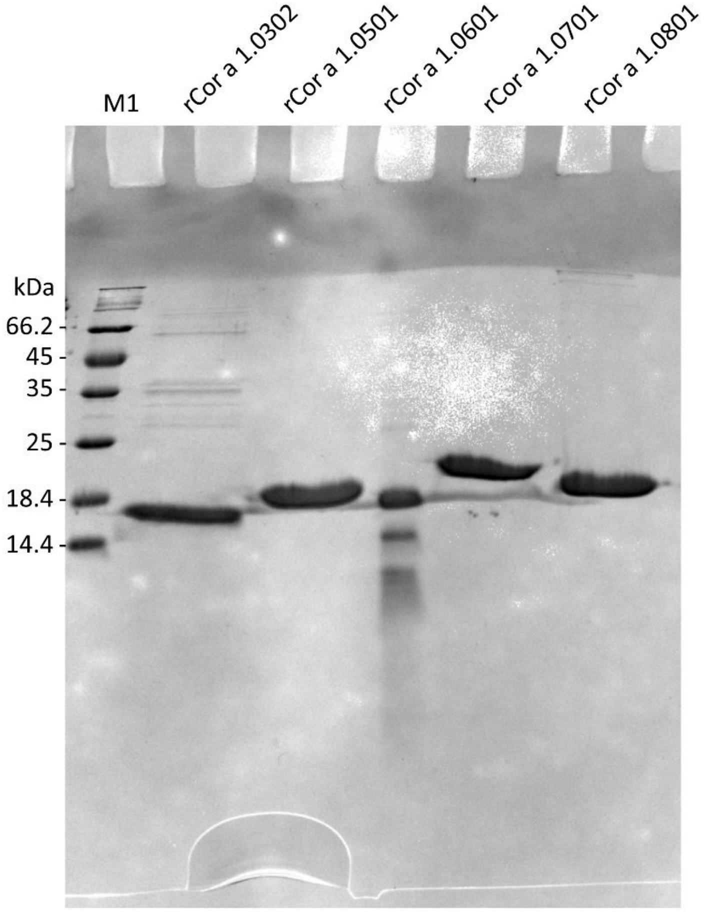


**Figure S4. Coomassie stained SDS-polyacrylamide gel showing Cor a 1 proteins purified from *E. coli*.** 5 µg of the purified proteins were applied on a 19 % SDS polyacrylamide gel and subjected to electrophoresis, followed by Coomassie staining. M1 molecular weight marker. The sizes of the marker proteins are indicated on the left.

## Figure S5

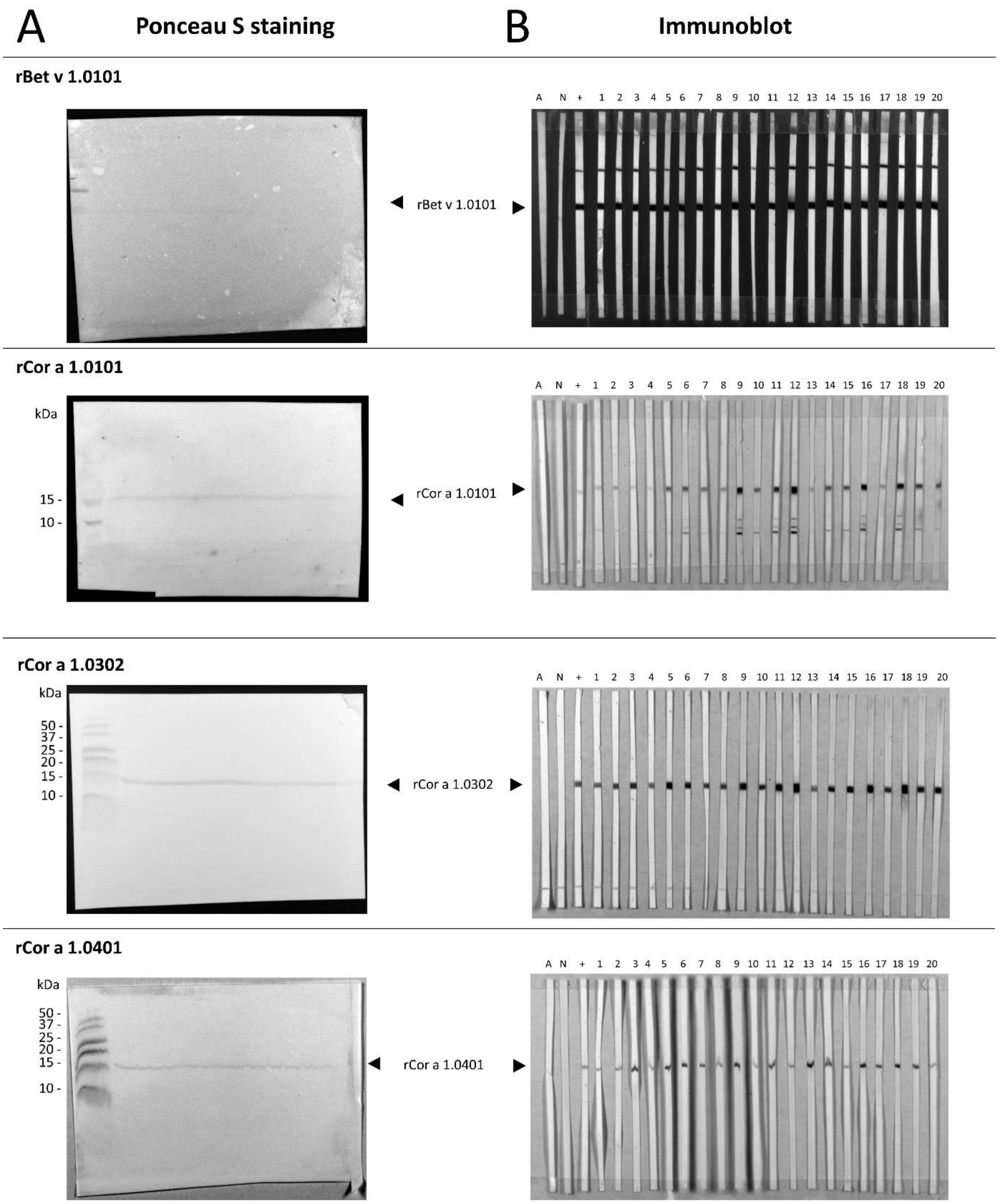

A
Ponceau S staining
B
Immunoblot
rBet v 1.0101
A N + 1 2 3 4 5 6 7 8 9 10 11 12 13 14 15 16 17 18 19 20
rBet v 1.0101
rCor a 1.0101
kDa
15 -
10 -
A N + 1 2 3 4 5 6 7 8 9 10 11 12 13 14 15 16 17 18 19 20
rCor a 1.0101
rCor a 1.0302
kDa
50 -
37 -
25 -
20 -
15 -
10 -
A N + 1 2 3 4 5 6 7 8 9 10 11 12 13 14 15 16 17 18 19 20
rCor a 1.0302
rCor a 1.0401
kDa
50 -
37 -
25 -
20 -
15 -
10 -
A N + 1 2 3 4 5 6 7 8 9 10 11 12 13 14 15 16 17 18 19 20
rCor a 1.0401

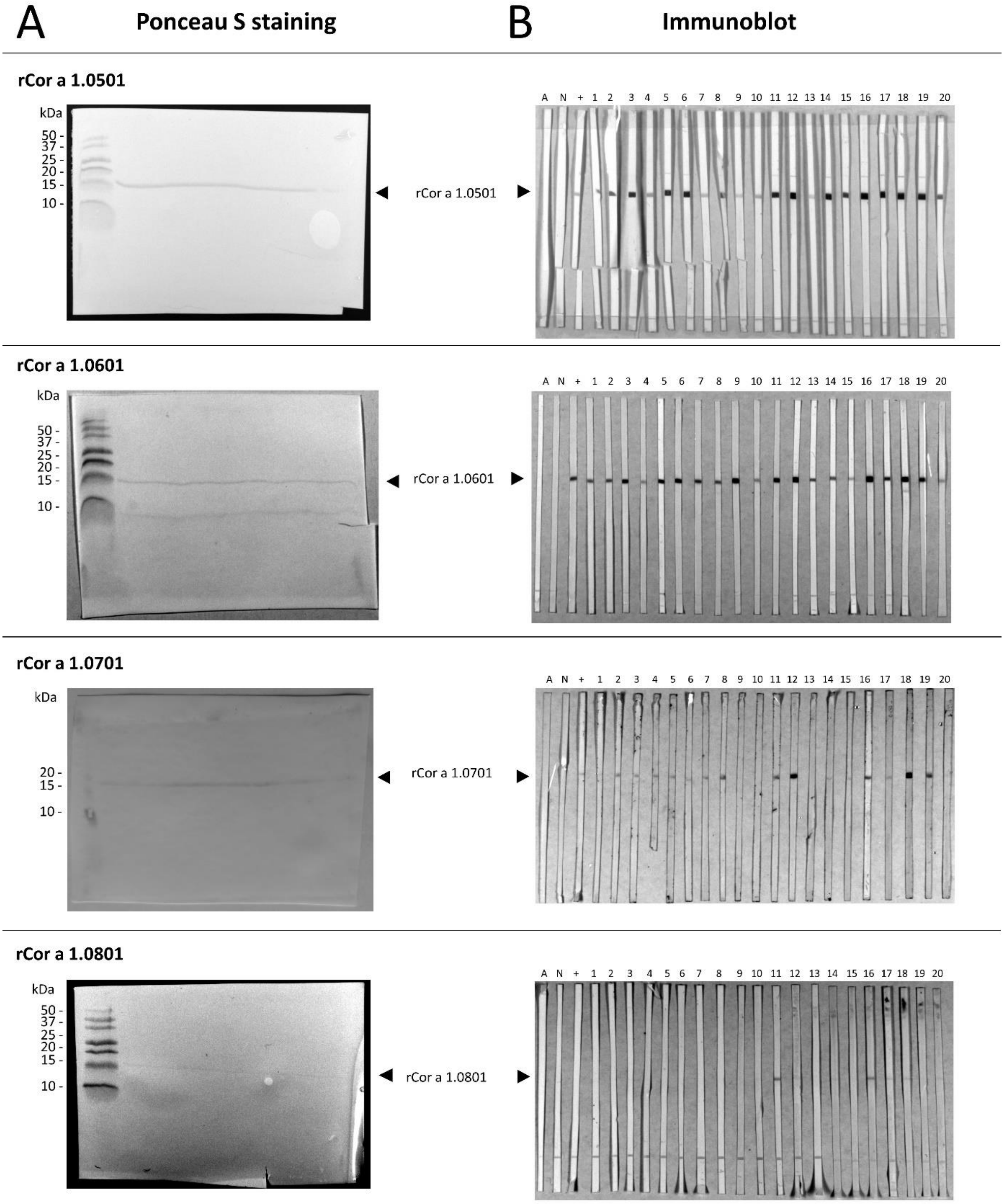


**Figure S5: Immunoblots of Cor a 1 isoallergens with sera from tree pollen allergic individuals. A) Ponceau S staining.** SDS-PAGE (16 % PA gels) was performed with 1.5 µg/cm or 0.8 µg/cm (Cor a 1.0801) of the Cor a 1 proteins shown, followed by blotting onto 0.2 µm nitrocellulose membranes. Successful protein transfer was visualized by Ponceau S staining. **B) Immunoblots**. The membranes produced in **A)** were cut into stripes and incubated with patients' sera. Bound specific IgE antibodies were detected using a mouse-anti-human IgE antibody coupled to alkaline phosphatase followed by nitroblue tetrazoliumchloride (NBT) /5- Bromo-4-chloro-3-indolylphosphate (BCIP) staining. Labels on top of the immunoblots: A, secondary antibody control; N, horse serum; +, positive control, mixture of sera from birch pollen and hazel pollen allergic persons (purchased from Gold Standard Diagnostics Kassel GmbH); 1-20, sera of patients allergic to tree pollen.

**Figure S6**

**A**

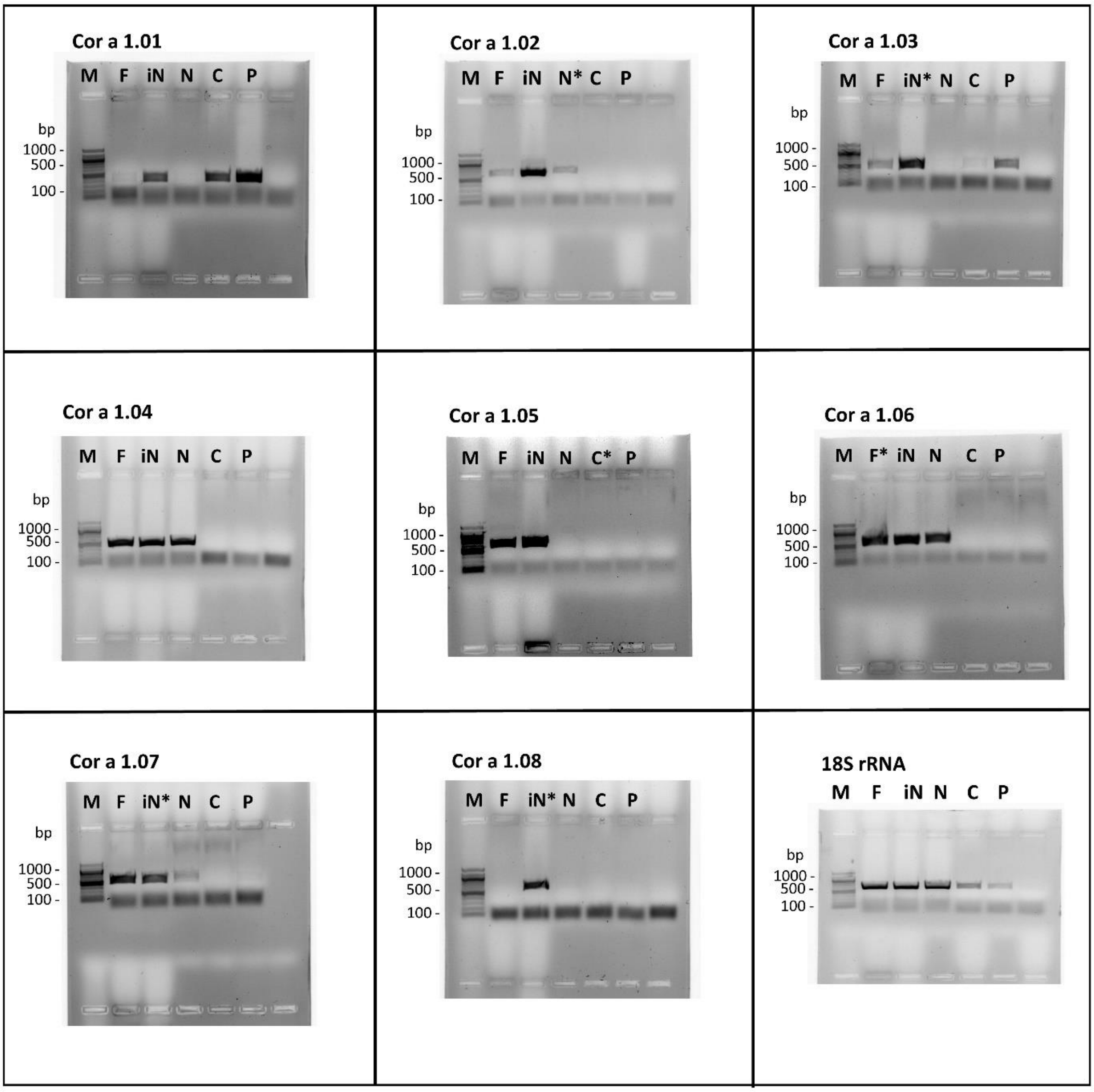

Cor a 1.01
M F iN N C P
bp
1000 -
500 -
100 -
Cor a 1.02
M F iN N* C P
bp
1000 -
500 -
100 -
Cor a 1.03
M F iN* N C P
bp
1000 -
500 -
100 -
Cor a 1.04
M F iN N C P
bp
1000 -
500 -
100 -
Cor a 1.05
M F iN N C* P
bp
1000 -
500 -
100 -
Cor a 1.06
M F* iN N C P
bp
1000 -
500 -
100 -
Cor a 1.07
M F iN* N C P
bp
1000 -
500 -
100 -
Cor a 1.08
M F iN* N C P
bp
1000 -
500 -
100 -
18S rRNA
M F iN N C P
bp
1000 -
500 -
100 -

**B**

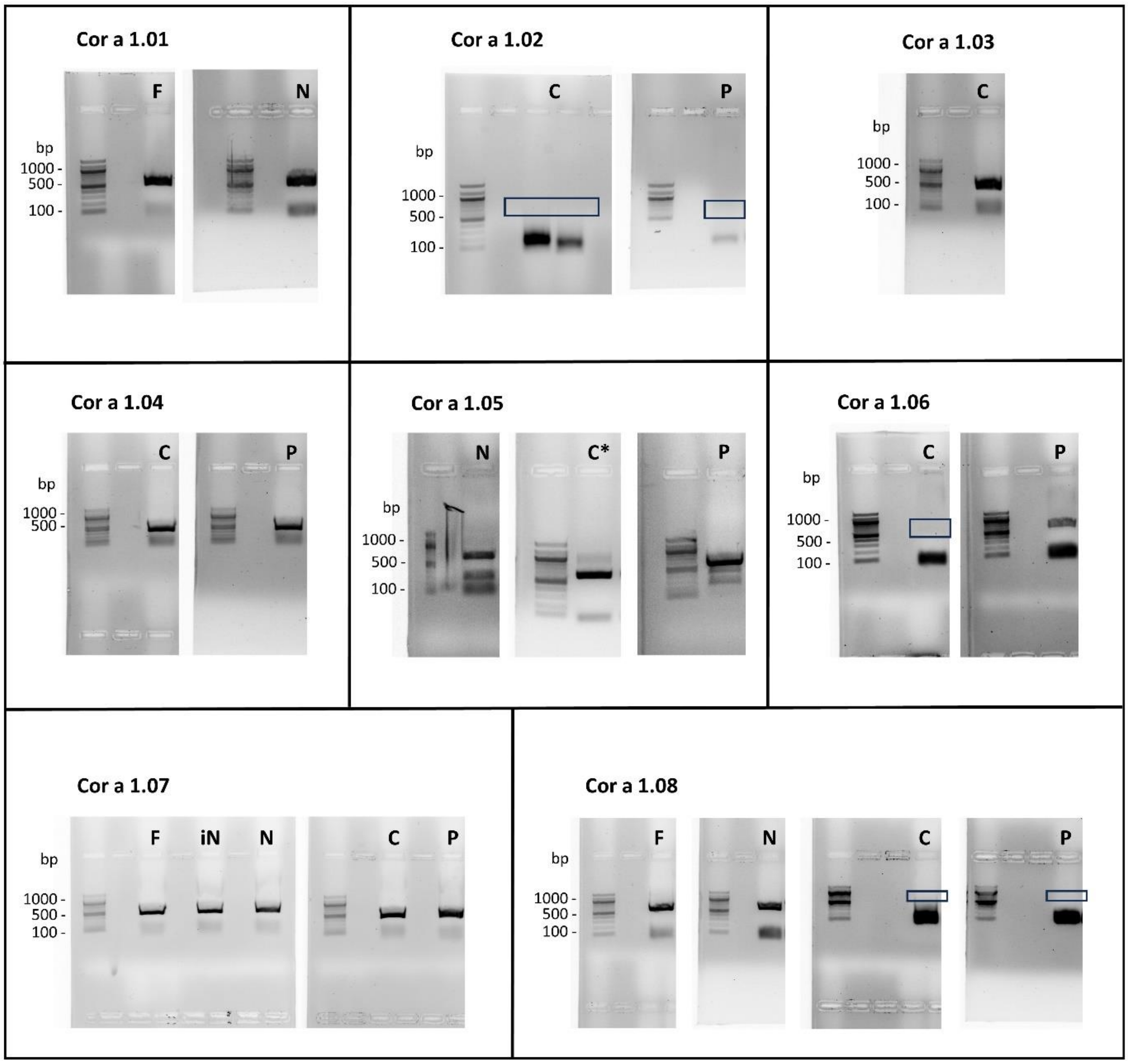


**Figure S6: Expression of Cor a 1 genes in different hazel tissues.** Full-length gels presented in Figure 1. The results of the 1st PCR assays are shown in A), the gels of the 2nd PCR assays are presented in B. The boxes indicate the regions where DNA products of the corresponding sizes were expected.

```
TTHA0849      --MPEVRAERYIPAPPERVYRLAK-DLEGLKP--YLKEVESLEVVAREGARTRSRWVAVA   55
Cor a 1.0801  MGVITYTDEYTSPIPPARLFKALVIDAHILIPKLLPQAVKSIEIIQGDGGAGSIRQITFA   60
                :     *   * ** *:::    * . * *    : *:*:*::  :*.    * ::.*

TTHA0849      MGKKVRWLEEEEWDDENLRNRFFSPEGDFDRYEGTWVFLPEGEGTRVVLTL---TYELTI  112
Cor a 1.0801  EGSQFSTVKNR-IDELNEKTYYY-------KYT---VIEFEGDALADKLELIVHEVQFEA  109
               *.:.  :::.  *: * :. ::       :*    *:  **:.    * *     ::

TTHA0849      PIFGGLLRKLVQKLMQENV----ESLLKGLEERVLAASS--------------        147
Cor a 1.0801  TAEGGSKNKMTTKYHTKDDVVIKEEEIKAGKEKVLGMYKVVEGYLIQNPGAYA        162
                 **  .*:. *   ::     *. :*. :*:**.  .
```

| Identity matrix | TTHA0849 | Cor a 1 | | | | | | | | |
|---|---|---|---|---|---|---|---|---|---|---|
| | | 0101 | 0201 | 0301 | 0302 | 0401 | 0501 | 0601 | 0701 | 0801 |
| TTHA0849 | 100 | 21.68 | 23.08 | 23.78 | 23.08 | 22.22 | 20.42 | 25.87 | 22.38 | 20.98 |

**Figure S7: Sequence comparison and identity matrix of TTHA0849 and Cor a 1 proteins.** (*) conserved amino acids; (:) conservative exchanges; (.) semi-conservative exchanges. Numbers in the identity matrix indicate the amino acid sequence identity in %.
